# Exclusive Breastfeeding, Diarrhoeal Morbidity and All-Cause Mortality in Infants of HIV-Infected and HIV Uninfected Mothers: An Intervention Cohort Study in KwaZulu Natal, South Africa

**DOI:** 10.1371/journal.pone.0081307

**Published:** 2013-12-02

**Authors:** Nigel C. Rollins, James Ndirangu, Ruth M. Bland, Anna Coutsoudis, Hoosen M. Coovadia, Marie-Louise Newell

**Affiliations:** 1 World Health Organization, Geneva, Switzerland; 2 University of KwaZulu-Natal, Durban, South Africa; 3 Africa Centre for Health and Population Studies, University of KwaZulu-Natal, Mtubatuba, South Africa; 4 University of Glasgow, Glasgow, United Kingdom; 5 University of the Witwatersand, Johannesburg, South Africa; 6 University College London, Institute of Child Health, London, United Kingdom; Hannover Medical School, Germany

## Abstract

**Introduction:**

Antiretroviral drug interventions significantly reduce the risk of HIV transmission to infants through breastfeeding. We report diarrhoea prevalence and all-cause mortality at 12 months of age according to infant feeding practices, among infants born to HIV-infected and uninfected mothers in South Africa.

**Methods:**

A non-randomised intervention cohort study that followed both HIV-infected and HIV-uninfected mothers and their infants until 18 months of age. Mothers were supported in their infant feeding choice. Detailed morbidity and vital status data were collected over the first year. At the time, only single dose nevirapine was available to prevent mother-to-child transmission of HIV.

**Results:**

Among 2,589 infants, detailed feeding data and vital status were available for 1,082 HIV-exposed infants and 1,155 HIV non-exposed infants. Among exclusively breastfed (EBF) infants there were 9.4 diarrhoeal days per 1,000 child days (95%CI. 9.12-9.82) while among infants who were never breastfed there were 15.6 diarrhoeal days per 1,000 child days (95%CI. 14.62-16.59). Exclusive breastfeeding was associated with fewer acute, persistent and total diarrhoeal events than mixed or no breastfeeding in both HIV-exposed infants and also infants of HIV uninfected mothers. In an adjusted cox regression analysis, the risk of death among all infants by 12 months of age was significantly greater in those who were never breastfed (aHR 3.5, p<0.001) or mixed fed (aHR 2.65, p<0.001) compared with those who were EBF. In separate multivariable analyses, infants who were EBF for shorter durations had an increased risk of death compared to those EBF for 5-6 months [aHR 2.18 (95% CI, 1.56-3.01); p<0.001].

**Discussion:**

In the context of antiretroviral drugs being scaled-up to eliminate new HIV infections among children, there is strong justification for financial and human resource investment to promote and support exclusive breastfeeding to improve HIV-free survival of HIV-exposed and non-exposed infants.

**Trial Registration: ClinicalTrials.gov NCT01948557; http://clinicaltrials.gov/**

## Introduction

In resource-limited settings, where child mortality due to diarrhoea, pneumonia and malnutrition is common, the dilemma of how HIV-infected mothers should feed their infants has to a large extent been resolved by the effectiveness of antiretroviral drugs (ARVs) to reduce the risk of postnatal transmission through breastfeeding[1-3]. In such settings, the World Health Organization (WHO) recommends that HIV-infected mothers receive ARVs and exclusively breastfeed (EBF) for six months and continue breastfeeding until at least twelve months while introducing complementary feeds from six months onwards[4]. 

Yet there remain challenges to scale-up access to these interventions and the support that would make them feasible. Programmatic questions related to the added value and feasibility of promoting and supporting EBF in the context of HIV are common. It is also questioned whether HIV-infected mothers receiving lifelong ARV treatment (ART) need to stop breastfeeding at 12 months. These questions highlight the uncertainty among health professionals and policy makers regarding the effectiveness of EBF and continued breastfeeding to improve survival of HIV-exposed infants, independent of any consideration of HIV infection.

As part of a study examining the effect of infant feeding practices on vertical transmission of HIV, we investigated the effect of exclusive breastfeeding by HIV-infected and HIV uninfected mothers on infant morbidity and mortality. Here we report diarrhoea prevalence and all-cause mortality over the first 12 months of life according to infant feeding practices in the first 6 months of life, among all infants born in a largely rural community in South Africa with high HIV prevalence. 

## Methods

The protocol for this trial and supporting TREND checklist are available as supporting information; see Checklist S1 and Protocol S1.

### Ethics statement

Signed, written consent was requested from all participating women and the study was approved by the biomedical research ethics committee of the University of KwaZulu-Natal.

### Study Design and Population

A non-randomised intervention cohort study – the Vertical Transmission Study - was conducted at two sites in KwaZulu Natal, South Africa to determine the effect of EBF on postnatal transmission of HIV[5]. HIV uninfected women were also recruited to determine the effectiveness of peer support interventions on rates of EBF[6]. A detailed description of the cohort and intervention, and related outcomes have been reported[5-8]. In brief, HIV-infected and HIV uninfected pregnant women were enrolled at nine antenatal clinics, 7 rural, 1 periurban and 1 urban, if they were 16 years of age or older, planned to stay in the study area for at least 3 months after delivery, and provided written informed consent. Mothers were counselled and supported in their preferred infant feeding practice for the first six months by community-based feeding counsellors and by study nurses at health facilities. HIV-infected mothers were counselled according to WHO recommendations at the time namely EBF for the first 6 months of life or replacement feeding (RF) based on home circumstances[9]. Infant feeding practices and morbidity data for every day of the preceding week, hospitalizations and attendances at health facilities were collected by an independent team of field monitors who visited weekly from birth to nine months of age. Mothers kept food-intake and morbidity diaries for use during the field-monitoring interview. Feeding counsellors and field monitors were unaware of the mothers’ HIV status. If a mother was not present for a counselling or monitoring visit, the respective study teams returned on up to 2 consecutive days. Feeding and morbidity data were also collected at scheduled monthly study visits at local health facilities from 1 to 9 months of age, and three monthly from 9 to 24 months of age.

The HIV status of HIV exposed infants was routinely determined by HIV RNA PCR (Nuclisens HIV-1 QT, Organon Teknika, Boxtel, the Netherlands, and Nuclisens EasyQ HIV-1, Biomerieux, Boxtel, the Netherlands) using dried blood spot samples (DBS) collected onto filter paper when infants were 6 weeks of age. DBS were also collected monthly but samples were only tested for HIV after the cohort study was completed. Final HIV status of the infants were included in the analyses.

At the time, only single dose nevirapine was available to prevent mother-to-child transmission of HIV (PMTCT); lifelong ART through the provincial health services became available from late 2004 for eligible mothers and infants (those with advanced HIV disease). 

### Definitions

Cumulative infant feeding practises were classified according to WHO definitions of exclusive, predominant, partial or never breastfed [10-12] and were determined only at the time of analysis. Mixed breastfeeding refers to both predominant and partial breastfeeding[11].

Diarrhoea days were determined by maternal recall of loose, watery or more frequent than normal stools[13]. If blood was mixed in with the stool even once, then the infant was considered to have dysentery. Diarrhoea events were defined as 2 or more consecutive diarrhoeal days separated by at least two consecutive diarrhoea-free days[14]. Acute diarrhoea was therefore defined as 2 diarrhoeal days or more but less than 14 days. Persistent diarrhoea was defined as 14 diarrhoeal days or more[15,16]. Each diarrhoeal event was treated as an independent event.

Infants were considered to be perinatally infected if the DBS sample taken at 6 weeks was positive, postnatally infected if the 6 week sample was negative and any subsequent sample was positive and ‘timing of infection unknown’ if the first sample was taken after 6 weeks and was positive.

### Statistical analysis

Analyses were restricted to singletons and first born twins. Feeding patterns were considered in the first 5 months of life only because HIV-infected women were counselled to stop all breastfeeding at that time. Feeding categories were established from the dataset by application of algorithms that first classified infant-feeding practices on every day of life and then measured the cumulative pattern from birth. Continuous data with a normal distribution were assessed with t test, Mann-Whitney test for non-normal distributions, two-sample tests of proportions or χ^2^ for categorical variables, and Fisher’s exact test if numbers were small. Kaplan-Meier method was used to estimate the time-to-diarrhoea event and survival by feeding mode. Cox regression analysis was used to investigate the effect of infant feeding practices on diarrhoea in a time-varying approach, adjusting for factors known to be associated with morbidity. The effect of infant feeding practices on infant mortality was assessed using Cox regression analysis adjusting for other factors associated with mortality. For variables with missing data, we included ‘unknown’ as a category in the model to retain overall denominators. For each child, the observation time was taken as time from birth until event (morbidity or mortality), date last seen or end of observation (6 months for morbidity and 12 months for mortality analysis) whichever came first. Diarrhoeal incidence was calculated as the number of events that occurred in the observation period (reported as per 1000 child days); prevalence of diarrhoeal days was calculated as the total number of diarrhoeal days reported in the observation period.

The effect of feeding practice on morbidity and mortality was investigated in a time-varying approach, adjusting for potential confounders. No other outcome measure was treated as a time-varying variable. There potential confounders were infant’s sex and HIV status, water source and enrolment clinic. Observation time was censored at change of feeding practice from EBF to predominant, partial or never breastfed reflecting the end of the former category and the start of the latter. Data on diarrhoeal events or days of diarrhoea of children that died were censored from the time of death.

We used Stata (Version 11.2; Stata Corporation, College Station, TX, USA) for analysis.

## Results

**Figure 1:**
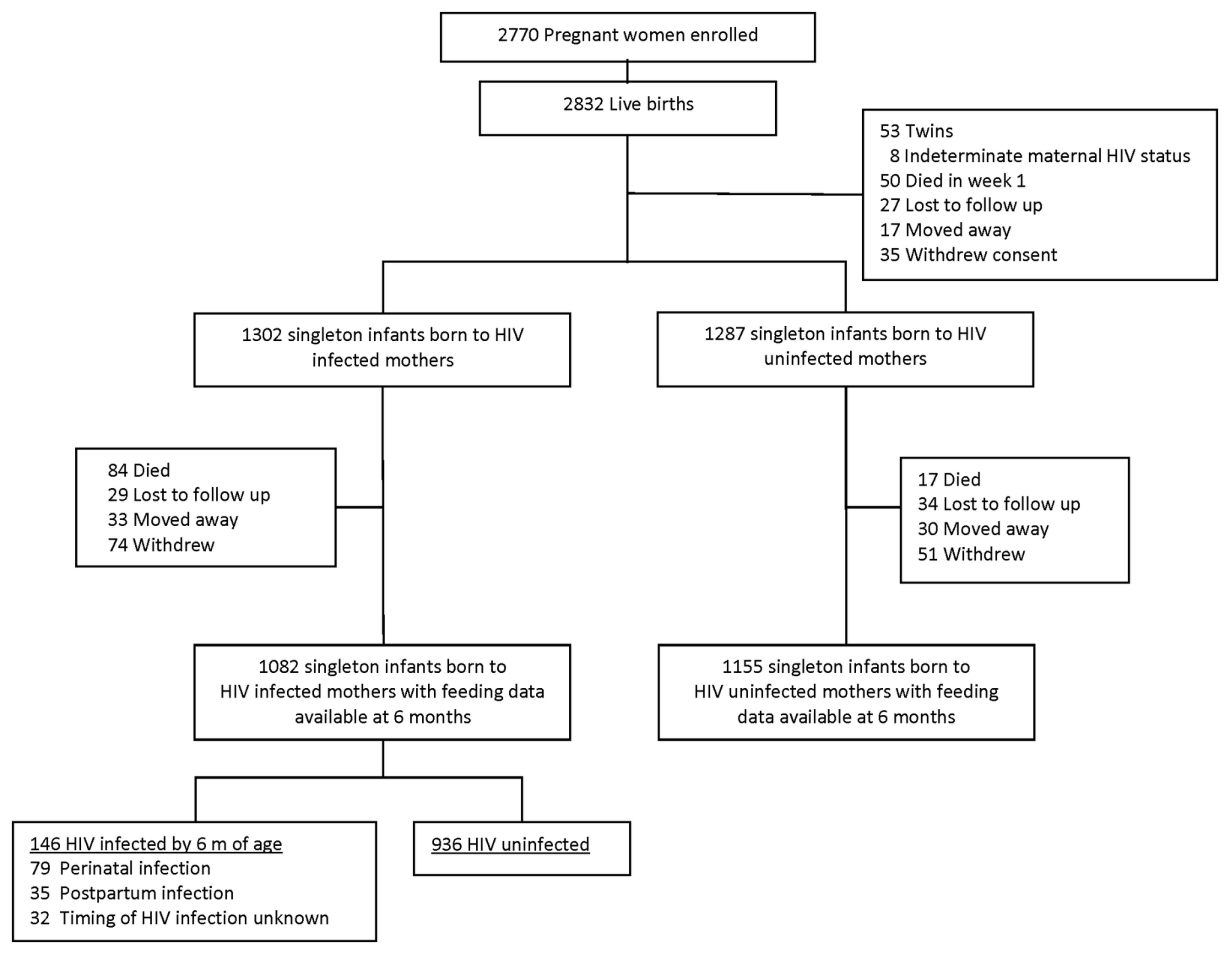
Cohort profile.

Between October 2001 to April 2005, 2,789 women gave birth to 2,832 infants. Characteristics and availability of infants for analysis are summarised in table 1 and Figure 1 respectively. Feeding data to 6 months of age were available for 1,155 singleton infants of HIV-uninfected mothers and 1,082 singleton infants of HIV-infected mothers and were therefore included in the analyses. Vital status was available for 2,502 infants and were included in the mortality analysis.

**Table 1:** Maternal and infant characteristics by maternal HIV status †

**Variable**	**HIV-infected mothers**	**HIV uninfected mothers**	**All**	p*
**Maternal age (n=2770)**				
years, median (IQR)	25.0 (21.5-29.4)	21.7 (19.0-27.9)	23.6 (20.0-28.9)	<0.001
**Maternal education (n=2770)**				
None	94 (6.7)	88 (6.4)	182 (6.6)	
1-9 years	516 (36.8)	454 (33.2)	970 (35.0)	
≥10 years	793 (56.5)	825 (60.4)	1,618 (58.4)	0.115
**Parity (n=2770)**				
0	471 (33.6)	686 (50.2)	1,157 (41.8)	
1-3	830 (59.2)	532 (38.9)	1,362 (49.2)	
≥4	102 (7.2)	149 (10.9)	251 (9.0)	<0.001
**Infants††**				
**Sex (n=2824)**				
Male (n, (%))	709 (49.4)	693 (49.9)	1,402 (49.6)	
Female	725 (50.6)	697 (50.1)	1,422 (50.4)	0.853
**Birth weight (n=2824)**				
≥2500 grams	1,166 (81.3)	1,203 (86.6)	2,369 (83.9)	
<2500 grams	157 (10.9)	103 (7.4)	260 (9.2)	
Missing	111 (7.8)	84 (6.0)	195 (6.9)	0.001
**Infant feeding practice (n=2,824)**				
Any breastfeeding	1,039 (72.5)	1,111 (79.9)	2,150 (76.1)	
Never breastfed	107 (7.4)	21 (1.5)	128 (4.5)	
Missing	288 (20.1)	258 (18.6)	546 (19.4)	<0.001
**Demographics (n=2824)**				
**Water source**				
Non-piped	428 (29.8)	402 (28.9)	830 (29.4)	
Piped	958 (66.8)	936 (67.3)	1,894 (67.1)	
Missing	48 (3.4)	52 (3.7)	100 (3.5)	0.762
**Enrolment clinic (n=2824)**				
Rural/Peri-urban	1,174 (81.9)	1,177 (84.7)	2,351 (83.3)	
Urban	260 (18.1)	213 (15.3)	473 (16.7)	0.046

^†^ Excluding mothers with indeterminate HIV status and their infants (n=8)

^††^ Data from both twins are included in the infant variables

^*^ statistical difference between HIV-infected and HIV-uninfected mothers

Among 2,097 infants who started EBF, the median time to cessation of all breastfeeding was 171 days (interquartile range: 82-180). Overall, 203 infants started RF of whom 47 were born to HIV uninfected mothers. Among HIV-infected and uninfected mothers, 81.4% and 92.9% were exclusively breastfeeding at 6-8 weeks of age and 61.8% and 72.6% at 3-4 months respectively; 3.4% and 2.3% were partial breastfeeding at 6-8 weeks of age and 14.1% and 15.8% at 3-4 months respectively; 15.2% and 4.8% were replacement feeding at 6-8 weeks and 24.1% and 11.6% at 3-4 months respectively.

### Diarrhoea prevalence in the first 6 months by feeding practice and infant HIV exposure/infection status

Among all infants there were 2,976 diarrhoeal days and 783 diarrhoeal events recorded in the first 6 months of infants’ lives. Among infants who were EBF in the first 6 months (n=1,399) there were 769 diarrhoeal days and 215 diarrhoeal events while among infants who were never breastfed (n=203) there were 196 diarrhoeal days and 59 diarrhoeal events. Among EBF infants this equated to 1.8 diarrhoeal events per 1,000 child days (95%CI. 1.70-2.01) and 9.4 diarrhoeal days per 1,000 child days (95% CI. 9.12-9.82) while among infants who were never breastfed this represented 2.5 diarrhoeal events per 1,000 child days (95%CI. 2.12-2.92) and 15.6 diarrhoeal days per 1,000 child days (95% CI. 14.62-16.59). Among the infants who were HIV exposed at birth there were 2.3 (95% CI 2.1-2.5) diarrhoeal events and 12.5 (95% CI 12.1-13.0) diarrhoeal days per 1,000 child days while among infants who were HIV unexposed at birth, there were 1.8 (95% CI 1.6-1.9) diarrhoeal events and 9.3 (95% CI 8.9-9.7) diarrhoeal days per 1,000 child days.

In unadjusted and adjusted analyses among all infants, EBF was associated with significantly fewer total diarrhoeal events in the first 6 months of life than mixed (predominant or partial breastfeeding) or no breastfeeding. (Table 2) The risks of acute and persistent diarrhoea were significantly higher in infants who were never breastfed compared to those who were EBF. Mixed feeding was associated with significantly greater risks for total and acute diarrhoea. Compared to infants born to HIV uninfected mothers, infants who were HIV-infected perinatally had significantly greater risk of total, acute and persistent diarrhoea. Infants who only became HIV-infected postnatally were more likely to have acute but not persistent diarrhoea. Infants born to HIV-infected mothers but who were themselves uninfected were not at increased risk of diarrhoea compared to HIV non-exposed infants. Other factors that increased the risk of diarrhoeal prevalence were male gender, non-piped water, rural or periurban enrolment clinic. Maternal age, parity or education had no effect in either unadjusted or adjusted analyses.

**Table 2:** Unadjusted and adjusted Cox regression of factors associated with diarrhoea morbidity in the first 6 months.

			**Overall diarrhoea**				**Persistent diarrhoea**				**Acute diarrhoea**			
	**Events/**		**Unadj HR**	**p**	**Adj HR**	**p**	**Unadj HR**	**p**	**Adj HR**	**p**	**Unadj HR**	**p**	**Adj HR**	**p**
	**Child-years**		**(95% CI)**		**(95% CI)**		**(95% CI)**		**(95% CI)**		**(95% CI)**		**(95% CI)**	
**Infant feeding practice**														
Exclusive breastfeeding		463/1186	1.00		1.00		1.00		1.00		1.00		1.00	
Predominant		16/18	1.43	0.049	1.38	0.080	1.51	0.278	1.37	0.407	1.40	0.097	1.38	0.122
Breastfeeding		157/327	(1.00-2.05)		(0.96-1.97)		(0.71-3.22)		(0.65-2.93)		(0.94-2.11)		(0.92-2.07)	
Partial breastfeeding		147/255	1.25	0.002	1.19	0.021	1.17	0.356	1.08	0.642	1.28	0.003	1.22	0.018
			(1.09-1.46)		(1.02-1.53)		(0.84-1.61)		(0.78-1.50)		(1.09-1.51)		(1.03-1.44)	
Never breastfed			1.26	0.001	1.35	<0.001	1.43	0.012	1.50	0.005	1.21	0.016	1.29	0.001
			(1.09-1.44)		(1.17-1.53)		(1.08-1.89)		(1.13-2.00)		(1.03-1.41)		(1.10-1.51)	
**Infant HIV status**														
HIV non-exposed		681/1158	1.00		1.00		1.00		1.00		1.00		1.00	
Perinatal infection or		63/483	1.46	<0.001	1.43	<0.001	2.01	<0.001	2.04	<0.001	1.29	0.022	1.27	0.037
unknown timing			(1.21-1.76)		(1.18-1.72)		(1.48-2.94)		(1.44-2.88)		(1.03-1.61)		(1.01-1.58)	
Postnatal infection		16/139	1.67	0.017	1.76	0.009	1.05	0.932	1.12	0.849	1.84	0.008	1.95	0.004
			(1.09-2.55)		(1.15-2.69)		(0.33-3.28)		(0.36-3.50)		(1.16-2.90)		(1.23-3.07)	
HIV exposed and		5/54	1.67	0.123	1.45	0.271	0.88	0.900	0.82	0.849	1.88	0.074	1.60	0.186
Uninfected			(0.86-3.22)		(0.75-2.79)		(0.12-6.27)		(0.36-3.49)		(0.94-3.78)		(0.79-3.21)	
Unknown status		18/22	0.31	<0.001	0.44	<0.001	0.33	0.029	0.44	0.108	0.31	<0.001	0.43	0.002
			(0.19-0.50)		(0.27-0.69)		(0.12-0.89)		(0.17-1.20)		(0.18-0.53)		(0.25-0.73)	
**Sex**														
Male		409/924	1.00		1.00		1.00		1.00		1.00		1.00	
Female		374/923	0.85	0.002	0.86	0.004	0.72	0.002	0.73	0.004	0.89	0.050	0.90	0.082
			(0.77-0.94)		(0.78-0.96)		(0.58-0.89)		(0.59-0.91)		(0.80-0.99)		(0.81-1.01)	
**Water Source**														
Non-Piped		289/613	1.00		1.00		1.00		1.00		1.00		1.00	
Piped		486/1216	0.70	<0.001	0.77	<0.001	0.83	0.101	0.91	0.466	0.67	<0.001	0.73	<0.001
			(0.63-0.77)		(0.69-0.86)		(0.66-1.03)		(0.73-1.15)		(0.59-0.75)		(0.65-0.82)	
Unknown		8/35.8	0.04	0.001	0.15	0.056	-		-		0.05	0.003	0.18	0.085
			(0.01-0.28)		(0.02-1.05)						(0.01-0.35)		(0.02-1.27)	
**Enrolment clinic**														
Rural/Peri urban		704/1615	1.00		1.00		1.00		1.00		1.00		1.00	
Urban		78/250	0.47	<0.001	0.52	<0.001	0.42	<0.001	0.43	<0.001	0.48	<0.001	0.55	<0.001
			(0.39-0.56)		(0.43-0.63)		(0.28-0.63)		(0.28-0.65)		(0.39-0.58)		(0.44-0.67)	

Diarrhoea events after EBF cessation were associated with the mode of feeding following EBF. Children who did not receive any breastmilk following EBF cessation had higher incidence of diarrhoea events than those who were mixed-fed, irrespective of duration of EBF. Among the children who were EBF to 6 months, the incidence of diarrhoea events per 1,000 child-days was 3.4 (95% CI. 2.6-4.4) if they received no breastmilk after EBF and 2.4 (95% CI. 2.1-2.7) if there was continued breastfeeding in addition to complementary foods following cessation of EBF. In multivariate analysis adjusting for feeding practice after EBF, infant gender and HIV status, infants who were EBF for 2-4 months had an increased risk of diarrhoea morbidity compared to those EBF for 5-6 months (AHR 1.35 [95% CI, 1.14-1.59]).

### Mortality by 12 months of age by feeding practice and infant HIV exposure/infection status

In an adjusted cox regression analysis, the risk of death among all infants by 12 months of age was significantly greater in those who were never breastfed (aHR 3.5, p<0.001) or mixed fed (aHR 2.65, p<0.001). (Table 3) Postnatal HIV infection through breastfeeding was associated with higher mortality compared to HIV non-exposed infants, while the risk of death associated with perinatal HIV infection was even higher at 13 times greater risk of death. Maternal education was associated with reduced infant mortality (AHR 0.52 [95% CI 0.32-0.85. p=0.01]) but other maternal characteristics such as age, parity were not associated with a reduced risk. 

**Table 3:** Risk factors associated with mortality in the first 12 months of life.

	**Events/ child-years**	**Unadjusted HR (95% CI)**	**P**	**Adjusted HR (95% CI)**	**P**
**Infant feeding practice**					
Exclusive breastfeeding	78/1449	1.00		1.00	
Predominant breastfeeding†	-	-	-	-	-
Partial breastfeeding	31/431	3.05	<0.001	2.65	<0.001
		(1.79-5.18)		(1.85-3.79)	
Never breastfed	60/366	21.62	<0.001	3.53	<0.001
		(13.52-34.58)		(2.44-5.12)	
**Infant HIV status**					
HIV non-exposed	58/2067	1.00		1.00	
Perinatal infection or	83/111	30.77	<0.001	13.16	<0.001
unknown timing		(18.01-52.61)		(9.00-19.22)	
Postnatal infection	14/27	7.99	<0.001	6.43	<0.001
		(3.47-18.38)		(3.46-11.96)	
HIV exposed, uninfected	2/8	1.30	0.453	0.77	0.253
		(0.65-2.58)		(0.49-1.21)	
Unknown status	12/115			6.11	<0.001
				(3.53-10.55)	
**Sex**					
Male	85/1164	1.00		1.00	
Female	84/1164	0.96	0.776	0.97	0.926
		(0.73-1.27)		(0.75-1.31)	
**Water Source**					
Non-Piped	56/711	1.00		1.00	
Piped	108/1575	1.06	0.705	1.19	0.285
		(0.78-1.45)		(0.86-1.65)	
Unknown	5/42	9.09	<0.001	5.87	<0.001
		(5.39-15.33)		(3.11-11.06)	
**Maternal HIV**					
HIV uninfected	29/1187	1.00		-	
HIV-infected	140/1138	3.07	<0.001	-	
		(2.24-4.21)			
**Enrolment clinic**					
Rural/Peri urban	152/1966	1.00		1.00	
Urban	17/362	0.64	0.052	0.66	0.081
		(0.41-1.00)		(0.42-1.05)	

^†^ There were no events among infants that were predominantly breastfed

**Figures 2:**
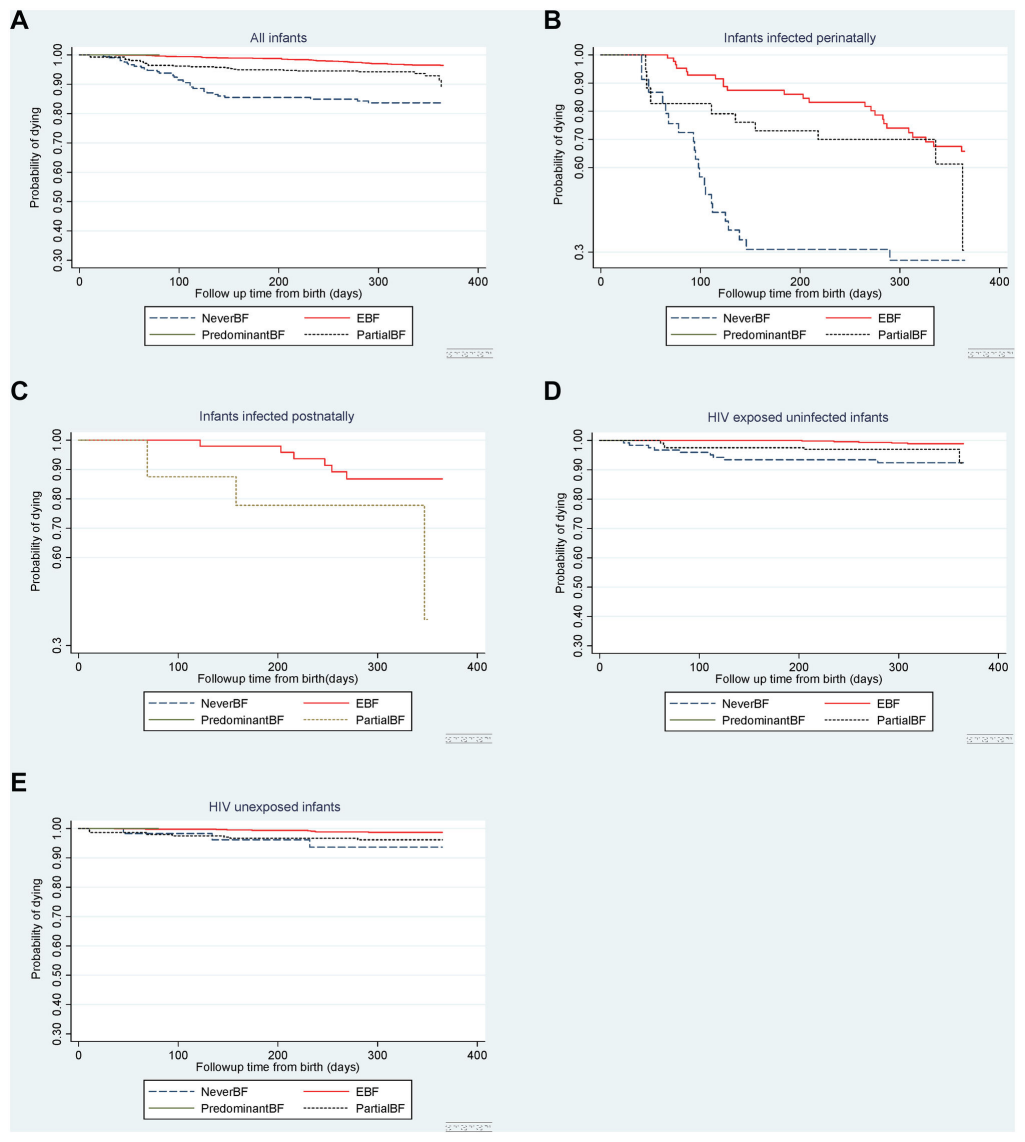
Survival by infant HIV exposure/infection status according to feeding practices in the first 6 months. a. All infants. 2b. Infants infected perinatally or timing unknown. 2c. Infants infected postnatally. 2d. HIV-exposed uninfected infants. 2e. HIV unexposed infants.

In multivariable analysis adjusted for infant HIV status and gender, parity, water source, enrolment clinic, maternal education and age, infants who were never breastfed had consistently higher mortality risk in all time intervals than infants who were exclusively breastfed. (Table 4) Infants who were mixed fed also incurred higher mortality. In a separate multivariable analysis, infants who were EBF for shorter durations had an increased risk of death compared to those EBF for 5-6 months [aHR 2.17 (95% CI, 1.58-2.98); p<0.001 for EBF 2-4 months and aHR 2.18 (95% CI, 1.56-3.01); p<0.001) for EBF under 2 months]. Children who had EBF up to 6 months had the lowest mortality rate compared to the other categories (Table 4). Figures 2 a-e shows Kaplan Meier analyses of the effect of infant feeding practices on mortality in the first 12 months of life according to cumulative infant feeding practice in the first 6 months.

**Table 4:** Multivariate analysis of breastfeeding practices and infant mortality in the first year of life *

	**Mortality in first 3 months**				**Mortality between 3-6 months**				**Mortality between 0-12 months**			
	**HR**	**p**	**AHR**	**p**	**HR**	**p**	**AHR**	**p**	**HR**	**p**	**AHR**	**p**
	**(95% CI)**		**(95% CI)**		**(95% CI)**		**(95% CI)**		**(95% CI)**		**(95% CI)**	
Exclusive breastfeeding	1.0		1.0		1.0		1.0		1.0		1.0	
Predominant breastfeeding†	-		-		-		-		-		-	
Partial	6.4	<0.001	2.8	0.008	1.9	0.113	2.6	0.113	3.9	<0.001	2.6	<0.001
breastfeeding	(3.5-11.7)		(1.3-6.1)		(0.9-4.1)		(0.8-8.4)		(2.9-5.4)		(1.9-3.8)	
Never	3.2	<0.001	3.4	<0.001	2.6	0.003	2.9	0.007	4.5	<0.001	3.6	<0.001
breastfed	(1.7-5.8)		(1.7-6.8)		(1.4-4.8)		(1.3-6.5)		(3.2-6.4)		(2.5-5.2)	

^*^ Adjusted for infant HIV status and gender, parity, water source, enrolment clinic, maternal education and age

^†^ There were no events among infants that were predominantly breastfed

There was no significant difference in mortality by feeding practice (continued breastfeeding or no breastfeeding) following EBF cessation. 

## Discussion

We present detailed infant diarrhoeal morbidity and all-cause mortality data from a high HIV prevalent rural and peri-urban setting in South Africa that highlight the major protection that exclusive breastfeeding provides to both HIV-exposed and non-exposed infants. In this study, both HIV-infected and uninfected mothers were supported to exclusively breastfeed and, with this support that included periodic home visits, the vast majority of mothers successfully did so. In settings such as South Africa where diarrhoea and pneumonia are still significant causes of under-5 mortality, and even where HIV is highly prevalent, promoting and supporting EBF at population level would reduce infant mortality in the entire population. The impact of improving EBF rates would be especially significant if ARV interventions that prevent postnatal HIV transmission are effectively scaled-up and provided to HIV-infected mothers who want to breastfeed.

The frequency of diarrhoeal events and burden of diarrhoeal days were significantly fewer among those infants who EBF compared to those who were never breastfed; this was true for both HIV exposed and non-exposed infants. EBF was also more effective at reducing diarrhoeal morbidity compared with either predominant or partial breastfeeding. The longer the duration of EBF, the greater was the protection. These reductions in diarrhoeal morbidity mirrored significantly lower mortality among EBF infants, both in the first months of life and also at 12 months of age. The increased mortality risk among infants who were never breastfed or only partially breastfed compared to those who EBF remained significant even when adjusted for HIV exposure and infection status. Environmental conditions such as water supply and rural setting were significant risk factors for diarrhoeal morbidity but not for infant mortality. Study population characteristics including maternal age, parity and education appeared to have no influence on diarrhoeal outcomes. However, higher level of maternal education was associated with a 48% reduction in infant mortality risk at 12 months.

Mothers were counselled to stop all breastfeeding around 6 months of age according to global recommendations at that time[9]. This involved explaining the risk of HIV transmission through continued breastfeeding and providing practical suggestions regarding transitioning from breastfeeding to an alternative milk source. No specific milk supplements were provided. Among HIV-exposed infants there was increased diarrhoeal morbidity in those infants whose mothers stopped all breastfeeding compared to those infants who continued to receive some breastmilk in addition to complementary feeds. While increased morbidity, growth faltering and mortality have been reported in other southern Africa settings where HIV-infected mothers stopped breastfeeding prematurely [17,18], there was no difference in mortality among HIV-exposed infants who stopped breastfeeding at 6 months compared to those who continued breastfeeding. However, this was most likely due to the limited number of events among infants followed-up.

The vast majority of infants breastfed for some period of time irrespective of whether they were HIV-exposed or not. Infants who were HIV-exposed but uninfected were neither at increased risk of diarrhoeal morbidity or death compared to infants born to HIV uninfected mothers. This suggests that these infants were either at no immunological disadvantage by virtue of their HIV exposure or that breastfeeding ameliorated any adverse consequence of being born to an HIV-infected mother. In contrast, infants who became HIV-infected during pregnancy or delivery were thirteen times more likely to die by 12 months of age compared to infants who were not exposed to HIV infection. However, infants who became HIV-infected postnatally were only about six times more likely to die in the first year suggesting that these infants managed to acquire some immunocompetency before becoming HIV-infected, and they had already survived the greatest risk period for infant mortality. While similar findings have been reported in recent meta-analyses [19], our data provide robust confirmation of this finding but from an infant population exposed to the same set of environmental conditions and risks.

The findings reported above are based on daily infant feeding and morbidity data that were collected weekly from HIV-infected and uninfected mothers at their homes in rural and periurban settings. Comparable datasets are not available elsewhere and the dataset permits valuable comparative analyses. Rigorous definitions of cumulative exclusive breastfeeding were strictly applied. HIV-infected mothers self-selected their infant feeding practice as it was deemed unethical and impractical to allocate practices by randomisation. Inherent differences among participating HIV-infected mothers that may influence morbidity and mortality outcomes have been previously reported in detail[20]. Mothers who chose to breastfeed tended to have poorer water supplies and sanitation, less income and less access to electricity. Despite these less favourable environmental circumstances, breastfeeding conferred a survival benefit on breastfed children. Diarrhoeal morbidity was inferred from maternal history and was only confirmed by the research team if the infant had loose stools at the time of home visit. Associations with lower respiratory infections were not examined in the analysis. For while data on cough and fast breathing were also collected, it is more difficult to apply and interpret clinical algorithms to estimate the prevalence of severe pneumonia and infer cause-specific deaths. 

In countries where national authorities promote and support breastfeeding while providing ARVs to HIV-infected mothers, WHO recommends that HIV-infected mothers exclusively breastfeed for the first 6 months and continue to breastfeed for at least the first 12 months while introducing complementary feeds around 6 months. While exclusive breastfeeding is reported to reduce the risk of postnatal HIV transmission compared to mixed feeding [5,21], in the context of ARVs that reduce the risk of HIV transmission exclusive breastfeeding makes a different and more significant contribution, namely to prevent other causes of infant mortality. Our findings support current WHO recommendations for improving HIV free survival of HIV exposed infants. WHO still recommends mothers who are HIV-uninfected to EBF until 6 months and continue breastfeeding until their infants are 24 months of age. Our findings confirm that infant mortality could be reduced by two or threefold if all infants were exclusively breastfed for 6 months[22]. 

Despite compelling evidence that EBF would make a significant contribution to reducing infant mortality in high HIV prevalence settings, and the consequences of not breastfeeding having been quantified in terms of mortality [22] and DALYs [23], national and district health authorities infrequently invest and aggressively promote exclusive breastfeeding among either HIV-infected or uninfected mothers. Health workers lack of confidence in their own ability to support exclusive breastfeeding and mothers doubt the merits and feasibility of EBF and continued breastfeeding. However a range of interventions including health policies [24], political leadership [25], systems interventions [26,27], simplified health facility [28] and community-based support [29,30], community engagement [31,32] and peer support [33] have reported improved exclusive breastfeeding rates under programmatic conditions.

The findings provide strong and further rationale for financial and human resource investment for promoting, supporting and protecting exclusive breastfeeding. In HIV prevalent settings such as South Africa, the evidence endorses a public health approach that actively invests in improving EBF rates while providing ARVs to HIV-infected mothers as linked child survival interventions – a direction that South Africa has moved towards through updated clinical protocols, introduction of legislation on the Code of marketing of Breastmilk Substitutes and new workplace regulations under consideration. The availability of simple ARV interventions to prevent postnatal transmission to infants, whether it be prophylaxis or lifelong treatment for breastfeeding HIV-infected mothers, creates the opportunity to harmonize and enhance interventions to improve infant feeding practices in the entire population. In this context, appropriate investments are essential to improve access and adherence to these regimens to ensure that benefits of breastfeeding for child survival are not undermined by inadequate ARV prophylaxis. The return on such financial investment, whether from HIV-designated funds or not, will not only increase the likelihood of HIV-free survival of HIV-exposed infants but also improve the health, development and survival of all children. 

## Supporting Information

Checklist S1 **Vertical Transmission Study TREND Checklist.**
(DOCX)

Protocol S1 **Vertical Transmission Study Protocol (v4.2).**
(DOC)
