# Supplementary material for: Exclusive Breastfeeding, Diarrhoeal Morbidity and All-Cause Mortality in Infants of HIV-Infected and HIV Uninfected Mothers: An Intervention Cohort Study in KwaZulu Natal, South Africa
Source: PLoS One. 2013 Dec 2;8(12):e81307. doi: 10.1371/journal.pone.0081307 (PMC3846835; doi:10.1371/journal.pone.0081307)
Supplement: Checklist S1 — Vertical Transmission Study TREND Checklist. (DOCX) [file pone.0081307.s001.docx]

**TREND checklist for**

**PONE-D-13-16166 [Exclusive breastfeeding, diarrhoeal morbidity and all-cause mortality in infants of HIV-infected and HIV uninfected mothers: An intervention cohort study in KwaZulu Natal, South Africa]**

| **Topic** | **Descriptor** | **Reported**  **Y/N** | **Section** |
| --- | --- | --- | --- |
| **Title and abstract** | Information on how unit were allocated to interventions | Y | Title and Abstract |
|  | Structured abstract recommended | Y | Abstract |
|  | Information on target population or study sample | Y | Title and Abstract |
| **Introduction** | Scientific background and explanation of rationale | Y | Introduction |
|  | Theories used in designing behavioral interventions | N |  |
| **Methods** |  |  |  |
| **Participants** | Eligibility criteria for participants, including criteria at different levels in recruitment/sampling plan (e.g., cities, clinics, subjects) | Y | Methods  Study design and Population |
|  | Method of recruitment (e.g., referral, self-selection), including the sampling method if a systematic sampling plan was implemented | Y | Methods  Study design and Population |
|  | Recruitment setting | Y | Study design |
|  | Settings and locations where the data were collected | Y | Study design |
| **Interventions**  *(see additional note at foot of table)* | Details of the interventions intended for each study condition and how and when they were actually administered, specifically including: | Y | Study design |
|  | o Content: what was given? | Y | Study design |
|  | o Delivery method: how was the content given? | Y | Study design |
|  | o Unit of delivery: how were the subjects grouped during delivery? | Y | Study design |
|  | o Deliverer: who delivered the intervention? | Y | Study design |
|  | o Setting: where was the intervention delivered? | Y | Study design |
|  | o Exposure quantity and duration: how many sessions or episodes or events were intended to be delivered? How long were they intended to last? | Y | Study design |
|  | o Time span: how long was it intended to take to deliver the intervention to each unit? | Y | Study design |
|  | o Activities to increase compliance or adherence (e.g., incentives) | N |  |
| **Objectives** | Specific objectives and hypotheses | Y | Introduction |
| **Outcomes** | Clearly defined primary and secondary outcome measures | Y | Introduction and Definitions |
|  | Methods used to collect data and any methods used to enhance the quality of measurements | Y | Study design |
|  | Information on validated instruments such as psychometric and biometric properties | N |  |
| **Sample size**  *(see additional note at foot of table)* | How sample size was determined and, when applicable, explanation of any interim analyses and stopping rules | N |  |
| **Assignment method** | Unit of assignment (the unit being assigned to study condition, e.g., individual, group, community) | Y | Study design and Statistical analysis |
|  | Method used to assign units to study conditions, including details of any restriction (e.g., blocking, stratification, minimization) | N/A |  |
| *(see additional note at foot of table)* | Inclusion of aspects employed to help minimize potential bias induced due to non-randomization (e.g., matching) | N |  |
| **Blinding (masking)** | Whether or not participants, those administering the interventions, and those assessing the outcomes were blinded to study condition assignment; if so, statement regarding how the blinding was accomplished and how it was assessed. | Y | Study design |
| **Unit of analysis** | Description of the smallest unit that is being analyzed to assess intervention effects (e.g., individual, group, or community) | Y | Statistical analysis |
|  | If the unit of analysis differs from the unit of assignment, the analytical method used to account for this (e.g., adjusting the standard error estimates by the design effect or using multilevel analysis) | N/A |  |
| **Statistical methods** | Statistical methods used to compare study groups for primary methods outcome(s), including complex methods of correlated data | Y | Statistical analysis |
|  | Statistical methods used for additional analyses, such as a subgroup analyses and adjusted analysis | Y | Statistical analysis |
|  | Methods for imputing missing data, if used | Y | Statistical analysis |
|  | Statistical software or programs used | Y | Statistical analysis |
| **Results** |  |  |  |
| **Patient flow** | Flow of participants through each stage of the study: enrollment, assignment, allocation, and intervention exposure, follow-up, analysis (a diagram is strongly recommended) | Y | Figure 1 |
|  | o Enrollment: the numbers of participants screened for eligibility, found to be eligible or not eligible, declined to be enrolled, and enrolled in the study | Y | Figure 1 |
|  | o Assignment: the numbers of participants assigned to a study condition | Y | Figure 1 |
|  | o Allocation and intervention exposure: the number of participants assigned to each study condition and the number of participants who received each intervention | Y | Figure 1 |
|  | o Follow-up: the number of participants who completed the follow-up or did not complete the follow-up (i.e., lost to follow-up), by study condition | Y | Figure 1 |
|  | o Analysis: the number of participants included in or excluded from the main analysis, by study condition | Y | Statistical analysis Figure 1 |
|  | Description of protocol deviations from study as planned, along with reasons | Y | Figure 1 |
| **Recruitment** | Dates defining the periods of recruitment and follow-up | Y | Results |

| **Baseline data** | Baseline demographic and clinical characteristics of participants in each study condition | Y | Table 1 |
| --- | --- | --- | --- |
|  | Baseline characteristics for each study condition relevant to specific disease prevention research | Y | Table 1 |
|  | Baseline comparisons of those lost to follow-up and those retained, overall and by study condition | N |  |
|  | Comparison between study population at baseline and target population of interest | Y | Table 1 |
| **Baseline equivalence** | Data on study group equivalence at baseline and statistical methods used to control for baseline differences | Y | Table 1 |
| **Numbers analysed** | Number of participants (denominator) included in each analysis for each study condition, particularly when the denominators change for different outcomes; statement of the results in absolute numbers when feasible | Y | Figure 1 Table 1 Table 2 Table 3 |
|  | Indication of whether the analysis strategy was “intention to treat” or, if not, description of how non-compliers were treated in the analyses | Y | Statistical analysis |
| **Outcomes and estimation** | For each primary and secondary outcome, a summary of results for each estimation study condition, and the estimated effect size and a confidence interval to indicate the precision | Y | 8 + Tables |
|  | Inclusion of null and negative findings | Y | Results |
|  | Inclusion of results from testing pre-specified causal pathways through which the intervention was intended to operate, if any | N |  |
| **Ancillary analyses** | Summary of other analyses performed, including subgroup or restricted analyses, indicating which are pre-specified or exploratory | N |  |
| **Adverse events** | Summary of all important adverse events or unintended effects in each study condition (including summary measures, effect size estimates, and confidence intervals) | N |  |
| **Discussion** |  |  |  |
| **Interpretation** | Interpretation of the results, taking into account study hypotheses, sources of potential bias, imprecision of measures, multiplicative analyses, and other limitations or weaknesses of the study | Y | Discussion |
|  | Discussion of results taking into account the mechanism by which the intervention was intended to work (causal pathways) or alternative mechanisms or explanations | Y | Discussion |
|  | Discussion of the success of and barriers to implementing the intervention, fidelity of implementation | Y | Discussion |
|  | Discussion of research, programmatic, or policy implications | Y | Discussion |

| **Generalisibility** | 21 | Generalizability (external validity) of the trial findings, taking into account the study population, the characteristics of the intervention, length of follow-up, incentives, compliance rates, specific sites/settings involved in the study, and other contextual issues | Y | Discussion |
| --- | --- | --- | --- | --- |
| **Overall evidence** | 22 | General interpretation of the results in the context of current evidence and current theory | Y | Discussion |

**Notes.**

A detailed account of the study methods, setting, enrolment approach, data collection tools and sample size are included either in the two methods paper published in the International Journal of Epidemiology and AIDS respectively or in the parent paper reporting the primary results of the study as designed i.e. HIV transmission results by feeding mode, namely

- Bland R, Coovadia H, Coutsoudis A, Rollins N, Newell M. Cohort profile: mamanengane or the Africa centre vertical transmission study. Int J Epidemiol 2010 April;39(2):351-60
- Bland RM, Little KE, Coovadia HM, Coutsoudis A, Rollins NC, Newell ML. Intervention to promote exclusive breast-feeding for the first 6 months of life in a high HIV prevalence area. AIDS 2008 April 23;22(7):883-91
- Coovadia HM, Rollins NC, Bland RM, Little K, Coutsoudis A, Bennish ML et al. Mother-to-child transmission of HIV-1 infection during exclusive breastfeeding in the first 6 months of life: an intervention cohort study. Lancet 2007 March 31;369(9567):1107-16.

These reports also describe measures taken to limit the effect of non-randomisation and non-blinding taken in the parent study. This included having separate teams for the counselling interventions and home-based collection of morbidity data and feeding practices, as well as corroboration against independently collected morbidity and feeding data collected at clinics.
