# Supplementary material for: Exclusive Breastfeeding, Diarrhoeal Morbidity and All-Cause Mortality in Infants of HIV-Infected and HIV Uninfected Mothers: An Intervention Cohort Study in KwaZulu Natal, South Africa
Source: PLoS One. 2013 Dec 2;8(12):e81307. doi: 10.1371/journal.pone.0081307 (PMC3846835; doi:10.1371/journal.pone.0081307)
Supplement: Protocol S1 — Vertical Transmission Study Protocol (v4.2). (DOC) [file pone.0081307.s002.doc]

# Africa Centre for Health and Population Studies, and

# The Department of Paediatrics and Child Health, University of Natal, Durban

The Vertical Transmission Study

**A cohort study to assess the impact of a breastfeeding counselling and support strategy to promote exclusive breastfeeding on post-natal transmission of HIV in African women**

Version 4.2

(including Amendments #1+#2+#3)

21^st^ May 2003

List of contents

[Investigators and sponsor 4](#__RefHeading___Toc530984944)

[Investigators 4](#__RefHeading___Toc530984945)

[Principal investigator 4](#__RefHeading___Toc530984946)

[Authorship 4](#__RefHeading___Toc530984947)

[Study site 4](#__RefHeading___Toc530984948)

[Sponsor 4](#__RefHeading___Toc530984949)

[Terms and abbreviations 5](#__RefHeading___Toc530984950)

[Summary for people with a scientific background 6](#__RefHeading___Toc530984951)

[Summary for lay people 6](#__RefHeading___Toc530984952)

[Introduction 7](#__RefHeading___Toc530984953)

[Background 7](#__RefHeading___Toc530984954)

[Aim 8](#__RefHeading___Toc530984955)

[Objectives 8](#__RefHeading___Toc530984956)

[Primary 8](#__RefHeading___Toc530984957)

[Secondary 8](#__RefHeading___Toc530984958)

[Hypothesis to be tested 8](#__RefHeading___Toc530984959)

[1. Study design 9](#__RefHeading___Toc530984960)

[1.1 General description 10](#__RefHeading___Toc530984961)

[1.2 Rationale for the study design 10](#__RefHeading___Toc530984962)

[2. Study site and population 11](#__RefHeading___Toc530984963)

[2.1 Study site 11](#__RefHeading___Toc530984964)

[2.2 Population 11](#__RefHeading___Toc530984965)

[2.3 Socio-demographic profile 11](#__RefHeading___Toc530984966)

[2.4 Current infant feeding practices 12](#__RefHeading___Toc530984967)

[3. Recruitment 12](#__RefHeading___Toc530984968)

[3.1 Inclusion and exclusion criteria 12](#__RefHeading___Toc530984969)

[3.2 Voluntary counselling and testing 13](#__RefHeading___Toc530984970)

[3.3 Enrolment 15](#__RefHeading___Toc530984971)

[3.4 Counselling on infant feeding options 15](#__RefHeading___Toc530984972)

[4. Antenatal care 16](#__RefHeading___Toc530984973)

[4.1 Initial history and examination 16](#__RefHeading___Toc530984974)

[4.2 Gestational age assessment 17](#__RefHeading___Toc530984975)

[4.3 Antiretroviral intervention (Nevirapine) 17](#__RefHeading___Toc530984976)

[4.4 Blood samples 18](#__RefHeading___Toc530984977)

[4.5 Folate and Iron 18](#__RefHeading___Toc530984978)

[4.6 Cotrimoxazole 18](#__RefHeading___Toc530984979)

[4.7 Home visits 18](#__RefHeading___Toc530984980)

[5. Delivery 18](#__RefHeading___Toc530984981)

[5.1 Obstetric practices 18](#__RefHeading___Toc530984982)

[5.2 Data collection 18](#__RefHeading___Toc530984983)

[5.3 Vitamin A supplements at delivery 19](#__RefHeading___Toc530984984)

[6. Post-natal home visits - Breastfeeding counselling and support strategy (BCSS) 19](#__RefHeading___Toc530984985)

[6.1 General description 19](#__RefHeading___Toc530984986)

[6.2 Breastfeeding support team 19](#__RefHeading___Toc530984987)

[6.3 BCSS schedule and procedures 19](#__RefHeading___Toc530984988)

[7. Post-natal home visits – Field monitoring 21](#__RefHeading___Toc530984989)

[7.1 General description 21](#__RefHeading___Toc530984990)

[7.2 Field monitoring team 21](#__RefHeading___Toc530984991)

[7.3 Field monitors schedule and procedures 21](#__RefHeading___Toc530984992)

[8. Post-natal clinic visits 22](#__RefHeading___Toc530984993)

[8.1 General description 22](#__RefHeading___Toc530984994)

[8.2 Study clinic team 22](#__RefHeading___Toc530984995)

[8.3 Clinic visit schedule, care and procedures 22](#__RefHeading___Toc530984996)

[9. Sampling methods, Diagnostic algorithms and Laboratory methods 24](#__RefHeading___Toc530984997)

[9.1 Sample collection and handling 24](#__RefHeading___Toc530984998)

[9.2 Determination of HIV status and CD4 count 25](#__RefHeading___Toc530984999)

[10. statistical analyses and Sample size calculation 26](#__RefHeading___Toc530985000)

[10.1 General considerations 26](#__RefHeading___Toc530985001)

[10.2 Assumptions re. transmission and feeding patterns for sample size calculation 26](#__RefHeading___Toc530985002)

[10.3 Study endpoints 27](#__RefHeading___Toc530985003)

[10.4 Statistical analyses 27](#__RefHeading___Toc530985004)

[10.5 Sample size calculation 29](#__RefHeading___Toc530985005)

[10.6 Interim analyses and study reviews 30](#__RefHeading___Toc530985006)

[11. Study management 31](#__RefHeading___Toc530985007)

[11.1 Study Steering Committee 31](#__RefHeading___Toc530985008)

[11.2 Data Monitoring and Safety Board (DMSC) 31](#__RefHeading___Toc530985009)

[11.3 Study discontinuation 31](#__RefHeading___Toc530985010)

[11.4 Confidentiality 31](#__RefHeading___Toc530985011)

[11.5 Biohazard risks 31](#__RefHeading___Toc530985012)

[11.6 Ethical approval 32](#__RefHeading___Toc530985013)

[11.7 Timelines 32](#__RefHeading___Toc530985014)

[Appendix A. Consent and Patient information forms 33](#__RefHeading___Toc530985015)

[Appendix B. UNAIDS recommendations on HIV and Infant feeding 38](#__RefHeading___Toc530985016)

[Risks of breastfeeding and replacement feeding 38](#__RefHeading___Toc530985017)

[Cessation of breastfeeding 38](#__RefHeading___Toc530985018)

[Infant feeding counselling 38](#__RefHeading___Toc530985019)

[Breast health 38](#__RefHeading___Toc530985020)

[Maternal health 38](#__RefHeading___Toc530985021)

[Appendix C. Statistical considerations regarding vertical transmission estimates 39](#__RefHeading___Toc530985022)

[References 40](#__RefHeading___Toc530985023)

# Investigators and sponsor

### Investigators

M Bennish Director, Africa Centre for Health and Population Studies,

Hlabisa District, Kwazulu/Natal.

R Bland Project paediatrician, Africa Centre for Health and Population

Studies, Hlabisa District, Kwazulu/Natal.

S Cassol Head of Africa Centre Virology laboratory, Durban.

HM Coovadia Professor of HIV Research, Nelson R Mandela School of Medicine

Research Institute, University of Natal.

A Coutsoudis Professor, Department of Paediatrics and Child Health,

University of Natal.

M-L Newell Professor in Epidemiology, Dept of Epidemiology and Public

Health, Institute of Child Health, London.

N Rollins Project leader, Department of Paediatrics and Child Health,

University of Natal and Africa Centre for Health and

Population Studies, Kwazulu/Natal.

G Solarsh Professor of Maternal and Child Health, Department of

Paediatrics and Child Health, University of Natal.

A Tomkins Professor, Centre for International Child Health, Institute of

Child Health, London.

### Principal investigator

Prof. HM Coovadia

### Authorship

Papers will be submitted by the principal investigators on behalf of the Child Health Group with individual contributions specifically identified.

### Study site

The Africa Centre for Health and Population Studies,

Mtubatuba, Hlabisa District, KwaZulu/Natal, South Africa.

### Sponsor

The Wellcome Trust (UK)

# Terms and abbreviations

| ACTG | Aids clinical trials group | | Research group who coordinate HIV related studies | | |
| --- | --- | --- | --- | --- | --- |
| ARV | Antiretroviral drug | | Drugs with properties to fight retroviruses | | |
| AZT | Zidovudine | | An antiretroviral drug of the reverse transcriptase inhibitor class | | |
| BCSS | Breastfeeding counselling and support strategy | | A comprehensive community-based strategy to promote exclusive breastfeeding | | |
| CAB | Community advisory board | | A representative group from the community and District Health Services who interact with the research centre to advise and comment on proposed and current research studies. | | |
| DBS | Dried blood spot | | Blood samples placed on filter paper for storage and analysis | | |
| DSA | Demographic surveillance area | | The geographic area under the surveillance of the DSS | | |
| DMSC | Data Monitoring and Safety Committee | |  | | |
| DSS | Demographic Surveillance System | | A research surveillance system to record vital statistics of the population e.g. migration, birth and mortality rates | | |
| DSS Home Tag |  | | An unique identifying number allocated to each home in the DSA | | |
| EBF | Exclusive breastfeeding | | Breastmilk only i.e. no water, formula milk etc. | | |
| ELISA | Enzyme linked immunosorbent assay | |  | | |
| HIV | Human immunodeficiency virus | |  | | |
| MTCT | Mother to child transmission | | Vertical infection of HIV from an HIV-infected mother to her child | | |
| EFF | Exclusive formula feeding | | Never received breast milk | | |
| MBF | Mixed breast feeding | | |  | |
| PCR | Polymerase chain reaction | |  | | |
| RCT | Randomised controlled trial | |  | | |
| SSC | Study Steering Committee | |  | | |
| VCT | Voluntary counselling and testing | | Pre-test and post-test counselling for HIV testing | | |
|  |  | | | |  |
| Breastfeeding counselling and support team (BCSS) | | The research community-based team responsible for implementing the breastfeeding counselling and support strategy. | | |  |
| Field monitoring team | | The research field team responsible for collecting data from mothers in their homes re. infant feeding practices. | | |  |
| Home supporter | | A member of the household, chosen by the mother, to participate in the study and offer support at home re. exclusive breastfeeding. | | |  |
| On-site rapid testing | | Rapid serological tests for the detection of HIV antibodies. | | |  |
| Study clinic assistant | | Member of study team based at participating clinics and hospitals. | | |  |
| Study HIV counsellor | | A trained HIV counsellor to offer VCT and enrol women into the study and also to provide ongoing support to mothers in the study. | | |  |

# Summary for people with a scientific background

Short course antiretroviral regimens such as AZT (Thai regimen), or nevirapine (Uganda HIVNET 012 regimen) given to HIV-infected women in pregnancy can reduce in utero and intrapartum transmission to their child by 40-50%. However, these gains are threatened by the continued transmission of HIV through breastmilk of poor HIV-infected women in Africa who have no realistic choice but to breastfeed. A recent study from Durban compared for the first time, mother to child transmission (MTCT) rates in each of the 3 feeding groups viz. exclusive breastfeeding (EBF), mixed breastfeeding (MBF) and formula feeding. The results of this study showed that at 3 months the MTCT in EBF mothers was similar to that in mothers giving only formula feeds, and the MTCT in EBF mothers was significantly lower than in those MBF (14.6% vs. 24.1%). This suggested that EBF may carry no additional risk of MTCT of HIV_1_ over formula feeding. A prospective study is required to verify these findings.

We propose a cohort study of HIV-infected mothers and their infants, to determine the rate of MTCT of HIV after mothers receive intensive breastfeeding counselling and support to practise EBF until about 6 months of age (the optimal duration for EBF). The main aims of the study will be to determine the effect of different infant feeding practices on HIV-infection rates of infants at 6 and 22 weeks and to determine the transmission incidence attributable to EBF. HIV-infected women will be counselled on infant feeding practices according to UNAIDS guidelines. All HIV-infected women and a random sub-sample of HIV-uninfected women will be recruited antenatally. Those who choose to breastfeed will be visited frequently at home by a team of breastfeeding counsellors before and after delivery to promote and support EBF. HIV-infected mothers who choose not to breastfeed will be supported in their choice by clinic-based staff. An independent team of field monitors will collect data weekly on feeding practices and morbidity of all infants at home visits. Blood samples will be collected by finger/heel prick and stored on filter paper prior to qualitative and quantitative estimation of HIV viral content to determine the timing of transmission.

# Summary for lay people

Short courses of drugs can be given to HIV-infected pregnant women to reduce the chance of HIV infection being passed to her child either during pregnancy or during the labour process. However, children can also become infected by drinking the mother’s breastmilk which contains the HIV virus. In many poor, developing countries in Africa, breastfeeding is the normal way of infant feeding and is vitally important because of the protection it gives to children from other diseases such as diarrhoea and malnutrition. Ideally there would be a way to make breastfeeding safer from HIV transmission without losing its other advantages.

A medical study recently suggested that HIV-infected women who exclusively breastfed their children i.e. gave breastmilk but without any water, tea, formula milk or any solid foods did not pass on the virus to their children to the same degree as women who MBF with these other fluids and foods. It is important to confirm whether this observation is in fact true or not.

We propose to follow 2,100 HIV-infected pregnant women and also some HIV-uninfected women from the time that they book at the clinic until 24 months of age. HIV-infected women who say they intend to breastfeed and all the HIV-uninfected women will be visited at their homes by breastfeeding counsellors both before and after delivery to support exclusive breastfeeding. HIV-infected women who choose not to breastfeed will be helped by clinic staff to safely replace all breastmilk with some other milk. An independent team will visit all mothers at their homes and collect information about the way they feed their children. Blood samples will be collected from the children at different times by a simple heel prick and the blood stored on a piece of filter paper. By testing these samples and comparing with the type of feeding at that time, we will be able to see when a child becomes infected and whether exclusive breastfeeding gives any protection.

# Introduction

South Africa is a society in the health transition, with infant mortality rates of 45 per thousand live births, substantially less than most other sub-Saharan African countries. The gains that have been made in infant and child mortality in the last decades, however, stand to be reversed by the HIV epidemic. At the Africa Centre field site in Hlabisa district of KwaZulu Natal, more than one-third of women coming for pre-natal care are infected with HIV.

Children are at risk from both vertically transmitted infection with HIV, and from the effects that HIV has on the health of parents and other adults in the community. Thus it is critical to better understand the increased health risks that children in rural South Africa now face. This grant application proposes to establish a mother-infant cohort of both HIV-infected and uninfected women that will allow a broad range of studies to be conducted that are critical to maternal and child health. This cohort will be superimposed on the existing Demographic Surveillance System at the Africa Centre in an attempt to achieve maximal utilisation of that Wellcome Trust-funded resource. It will provide the capability to study in detail the impact of the HIV epidemic on young children, and also to test key interventions to both prevent HIV infection and to improve the lives of those children already affected. The first proposed study, described in detail below, will examine risk factors for post-natal transmission of HIV, and interventions to reduce post-natal transmission of HIV from HIV-infected mothers to their children through breast milk.

# Background

Short and relatively simple antiretroviral (ARV) regimens have been recently reported to substantially reduce peripartum mother to child transmission (MTCT) of HIV. These significant gains, however, are threatened by the continued exposure of young infants to HIV-infected breast milk and the risk of post-natal transmission. Zidovudine (AZT) given to non-breastfeeding American and French women according to the ACTG 076 trial regimen achieved a reduction of MTCT of HIV of 67%.^1^ Shorter ARV regimens such as that used in the Thai study, and which are more suited to developing countries, have decreased MTCT by ~40 to 50%.^2-7^ However, follow-up in West African studies has found that in women who breastfeed the efficacy of the ARV intervention declines to 30% at 15 months^6^ and 23% at 24 months.^4^ A single study in Uganda (HIVNET 012),^8,9^ which gave one dose of nevirapine (NVP) to the mother at the onset of labour and to her baby soon after birth, gave a 50% decrease in MTCT at 6 weeks and a 42% decrease at 12 months when compared to a short course of AZT. In the PETRA study, a multicentre study conducted at 5 different sites in Africa, an even greater reduction of efficacy due to breastfeeding was evident at 18 months.^10^ The South African nevirapine trial (SAINT) comparing NVP (mother - intrapartum + mother and child - day 2 or 3) with AZT and lamivudine (mother - intrapartum + mother and child - 1 week post partum) demonstrated 13.6% transmission compared with 22.1% transmission at 14-16 weeks respectively.^11^ Although difficult to quantify, some of the ARV regimens used in the trials referred to above, especially those with a post-natal component,^3,7,8,9^ may reduce early breastfeeding transmission of HIV.

Breastfeeding accounts for about a third to half of all perinatal HIV transmission among breastfeeding women. Varying sensitivity of the tests used to detect HIV has introduced some imprecision in estimating the timing of vertical transmission. Among formula fed babies who are negative for HIV by PCR soon after birth, evidence of HIV infection can be demonstrated up to approximately 6 weeks of age. Dunn et al suggest that a single PCR at 4 weeks of age has greater than 95% sensitivity for diagnosis of HIV infection and nearly all intrapartum transmission may be detectable by 4 weeks in non-breastfed infants.^12^ Among breastfed babies there may be overlap between intrapartum and breastmilk transmission in the first 6 weeks.

A number of suggestions – including wet nursing, heat treatment of expressed milk or allowing expressed breastmilk to stand at room temperature have been put forward as ways to make breastfeeding safe;^13^ none can be easily accomplished in the conditions that prevail in rural South Africa. Several studies reported that 70-90% of African women who were appropriately counselled on the implications of breast or formula feeding chose breastfeeding.^3-9^ Breastmilk avoidance altogether or shortened periods of breastfeeding are the only real alternatives; Therefore there is an urgent need to explore the circumstances which would optimise the benefits and reduce the virus transmission risks of breastfeeding by HIV infected women in developing countries.

Our group recently compared, for the first time, MTCT rates in each of 3 feeding groups; exclusive breastfeeding (EBF) for at least 3 months, partial or mixed breastfeeding (MBF) and exclusive formula

feeding (EFF).^14^ At 6 months the MTCT in EBF mothers was the same as that in mothers giving only formula feeds (19.4% [95% CI 11.9-26.9] vs. 19.4% [95% CI 13.1-25.6]); MTCT in EBF mothers was significantly lower than in those who MBF (26.1% [95%CI 20.3-31.8]). This suggested that EBF may carry no additional risk of MTCT of HIV over formula feeding. However, these unexpected but promising findings were obtained from a trial whose main purpose was to test the efficacy of vitamin A in reducing MTCT of HIV.^15^ In the Thai and ACTG 076 studies mothers gave formula feeds. In the West African studies the type of breastfeeding was not specified but it is assumed (based upon known feeding patterns) that the method was MBF. This assumption is based on the following: with few exceptions, the majority of women described in the national surveys do not EBF; in more detailed research studies on feeding conducted in Mexcio^16^, South Africa^17^, USA^18^ and Bangladesh,^19,20^ EBF is practised by a minority of women. It is difficult to envisage any selective pressures which would induce HIV-infected pregnant women in the studies referred to above to breastfeed differently from this pattern. A pilot study that we have conducted at the Africa Centre on breastfeeding practices among women in Hlabisa district confirms the dominance of MBF from birth.

Accordingly, the results of the study that we have conducted in Durban need to be assessed in a study that has as its primary objective determining the relationship between feeding pattern and MTCT of HIV.

# Aim

To examine the risk of post-natal transmission of HIV in relation to infant feeding practices.

# Objectives

### Primary

1. To determine the effect of infant feeding practices on HIV infection rates of infants at 6 and 22 weeks of age.
2. To determine the infant survival rate at 24 months of age according to feeding practices and HIV status.

### Secondary

1. To determine the HIV infection rate of infants as measured of a sample collected within 72 hours of birth.
2. To determine the HIV transmission incidence attributable to the duration of different feeding practices.
3. To determine the cumulative incidence of vertical transmission in EBF, MBF and EFF infants.
4. To describe risk factors, other than feeding practice, for post-natal transmission of HIV, including maternal and infant morbidity and breast health.
5. To assess the determinants of transmission in MBF adjusting for exposure factors e.g. type and age of introduction of other food/milk.
6. To describe the morbidity and growth of infants in relation to feeding practices and HIV status.
7. To describe adherence rates to EBF following a breastfeeding support intervention.

# Hypothesis to be tested

Exclusive breastfeeding by HIV-infected women carries no additional risk of post-natal transmission of HIV than exclusive formula feeding.

**Study Design and Methods**

# 1. Study design

Table 1. Summary of procedures and investigations

|  | 1^st^ A/N visit | 2^nd^ A/N visit | | 28-32 wks gest | | 34 wks gest | <72 hrs | 6 wk | | 10 wk | | 14 wk | 18 wk | 22 wk | 26 wk | | 7 mt | 8 mt | 9 mt | 12 mt | 15 mt | 18 mt | 24mt |
| --- | --- | --- | --- | --- | --- | --- | --- | --- | --- | --- | --- | --- | --- | --- | --- | --- | --- | --- | --- | --- | --- | --- | --- |
| Consent for HIV testing | X |  | |  | |  |  |  | |  | |  |  |  |  | |  |  |  |  |  |  |  |
| Consent to enrol |  | X | |  | |  |  |  | |  | |  |  |  |  | |  |  |  |  |  |  |  |
| Counsel on infant feeding |  |  | | X | |  |  |  | |  | |  |  | X | X | | X | X | X |  |  |  |  |
| Maternal history and examination |  | X | |  | |  |  |  | |  | |  |  |  |  | |  |  |  |  |  |  |  |
| Dispense Maternal NVP |  |  | |  | | X |  |  | |  | |  |  |  |  | |  |  |  |  |  |  |  |
| Maternal NVP |  |  | |  | |  | X |  | |  | |  |  |  |  | |  |  |  |  |  |  |  |
| Infant NVP |  |  | |  | |  | X |  | |  | |  |  |  |  | |  |  |  |  |  |  |  |
| First antenatal home visit |  |  | | X | |  |  |  | |  | |  |  |  |  | |  |  |  |  |  |  |  |
| BCCS | Antenatal schedule | | | | | | Weekly and two weekly visits as per  the field schedule (See 6.3.3) | | | | | | | | |  | | | | | | | |
| Feeding monitoring |  | |  | |  |  | Weekly monitoring visits as per the field schedule (See 7.3.3) | | | | | | | | | | | | | | | | |
| Clinic visit – Nurse examination |  |  | |  | |  |  | | X | | X | X | X | X | X | | X | X | X | X | X | X | X |
| Clinic visit – Clinician examination |  |  | |  | |  |  | | X | |  |  |  |  |  | |  |  |  |  |  |  |  |
| Blood sampling – mother | X | X | |  | |  |  | | X | |  |  |  | X |  | |  |  |  |  |  |  |  |
| Blood sampling – infant * |  |  | |  | |  | X | | X^†^ | | X | X | X | X^†^ | X | |  |  | X | X | X | X | X |
| Breastmilk samples – mother |  |  | |  | |  | X | | X | | X | X | X | X | X | |  |  |  |  |  |  |  |

* Samples collected only if real-time test of previous sample at 6 and 22 weeks was negative for HIV

^†^ Sample processed real-time

## 1.1 General description

We propose to conduct an implementation study of enhanced support for EBF in a rural African community. We consider the main outcome measure to be the accumulation of HIV infections between 6 weeks (after which virtually all HIV transmission can be attributed to the feeding practice) and 22 weeks in infants of women who have been counselled and supported in EBF. HIV infection rates in infants who EBF and those who MBF will also be compared, as will the HIV infection rate in infants who EBF and the small number who are expected to EFF.

All pregnant women and mothers, HIV-infected and uninfected, will receive the same general infant feeding recommendation from the local health clinics i.e. EBF. After HIV testing, all HIV-infected women will be counselled regarding feeding options according to recent UNAIDS recommendations (Appendix B). All HIV-infected women will be invited to participate in the study and those who choose to breastfeed will receive intensive breastfeeding counselling and support at their homes for the first 26 weeks of life. HIV-infected women who choose to EFF will be supported by clinic-based staff and given appropriate advice for safe preparation and storage of formula milk. In addition, a smaller cohort of HIV-uninfected women will be recruited and receive the same breastfeeding counselling and support. The breastfeeding counselling message and support will be independent of HIV status and field-based breastfeeding counsellors will be blinded to the HIV status of all participants.

All mothers will be visited weekly by an independent team of feeding monitors to document actual feeding practices over the first 26 weeks of life or until breastfeeding has ceased. All mothers and children will also be followed up at regular clinic visits until 24 months of age. At these visits routine anthropometric assessments, blood and breastmilk samples and morbidity data will be collected from mothers and children.

2,100 HIV-infected and 400 HIV-uninfected women will be recruited. The HIV-uninfected group will be recruited to protect the confidentiality of the HIV-infected mothers, to determine the morbidity, growth and development in relation to infant feeding practices and to compare the adherence of HIV-infected and uninfected mothers to EBF. The breastfeeding support strategy will be evaluated to determine the most effective components of the intervention.

## 1.2 Rationale for the study design

We require a study design which is best suited to test our hypothesis that EBF carries minimal additional risk of transmitting HIV in the post-natal period. We argue against a randomised controlled trial on ethical and operational grounds. Rather, adopting the approach of an implementation study will offer valuable transmission data and programmatic information regarding breastfeeding promotion.

Although EFF averts all risk of post-natal transmission, a study design which includes randomisation to EFF in a community in which >90% initiate breastfeeding may expose the HIV-infected women to serious social risks due to the disclosure of HIV status. In Africa and in much of Asia, where breastfeeding has deep historical roots and profound cultural meaning, a shift to formula feeding is certain to produce major hardships. Formula feeding in a poor rural African community will also expose infants to high morbidity from causes other than HIV, and increase the likelihood of death from pneumonia, diarrhoeal disease, and other infectious diseases.^21^ The difficulties of a randomised trial are evident from a recent study in Kenya that attempted to randomise HIV-infected women to breastfeeding or formula feeding.^22^ Women in the breastfeeding group were encouraged to EBF but this was not monitored and so the actual practice is not known. Compliance of women randomised to formula feeding was only 70%, whereas 96% of those randomised to breastfeeding continued to do so. Running water and water-borne sanitation were normally available to this population. The women had 8 years of schooling and considerable support to give formula only, including the provision of formula milk. The accumulation of HIV-infected cases throughout the 24 months of study in the EFF group indicates the immense difficulties experienced in compliance; mortality due to diarrhoeal disease was higher in the HIV-uninfected, EFF children, especially in the first 6 months of life. The study took 7 years to complete in an area with an HIV prevalence of ±14% and in part presumably reflected the reluctance of women to be randomised.

MBF is unethical for randomisation in any study as it is our view that the limited evidence, supported by theoretical causal explanations, suggests this form of breastfeeding is accompanied by high MTCT rates. As MBF is the dominant mode of infant feeding it will be difficult to achieve adherence to EBF even after counselling and support. It is anticipated that even among women counselled to EBF, many will progressively switch to MBF.

We propose to implement a breastfeeding counselling and support strategy to promote EBF to women who choose to breastfeed. In a research setting with adequate community preparation, coupled with individual counselling and support, we hope to maintain EBF in sufficient numbers of mothers to achieve the objectives of this study. There are precedents to suggest that this is possible. In Mexico, peer counselling achieved 50% EBF for 3 months in women living in inner-city communities.^16^ Haider et al, in Dhaka, Bangladesh showed that 75% of mothers were EBF their infants two weeks after a hospital-based breastfeeding intervention, compared with 8% of mothers who did not receive the intervention.^19^ In another randomised controlled trial of peer counsellors in Dhaka, where most deliveries are at home, two home visits are made during the third trimester of pregnancy and then three more within the first 10 days of delivery; thereafter monthly visits are made. The objective of this ongoing study is to promote EBF for 5 months in communities that traditionally give pre-lacteal feeds, water and complementary feeds from birth. Preliminary results from this study indicate 85% of mothers in the intervention group are EBF at 4 days postpartum, compared to 30% of control mothers.^20^ Differences of similar magnitude continue to be seen during follow-up. Peer counselling also significantly increased rates of breastfeeding amongst a low-income population in the US Women, Infants and Children Program.^18^

Our experience in the Vitamin A intervention study, unsurprisingly, is that adherence is likely to be greater if women self-select feeding method.^15^

# 2. Study site and population

## 2.1 Study sites

The study will be conducted at two sites – Hlabisa sub-district which is a rural district in northern KwaZulu Natal and in Durban. In Hlabisa, there are 14 government clinics and one district hospital. The study will be implemented at 9 clinics within two tribal areas in the Hlabisa District of KwaZulu Natal Province. The study will be phased in over a 4 month period with recruitment starting at two clinics. Once established at these clinics another two clinics will be added. In Durban the study will be implemented at Kwadabeka clinic and St. Mary’s hospital, Mariannhill which is delivery site for many women who attend Kwadabeka for antenatal care.

The local health service providers in Hlabisa have secured funding from the Pediatric Aids Foundation ‘Call to Action’ programme to offer HIV counselling and testing to all pregnant women at the 14 government clinics in the district and to provide nevirapine to all women who are HIV-infected - the District Prevention of Mother to Child Transmission (PMTCT) Programme. The programme manager for the district programme, works in close collaboration with the vertical transmission study team.

## 2.2 Population

The Mpukunyoni and Hlabisa Tribal Areas of the Hlabisa Health District have a population of ~160,000 persons, with an expected 4-5,000 pregnancies per year. Anonymous antenatal surveillance found that ~30% of all women attending the antenatal services are HIV-infected. We thus estimate there will be 1200-1400 HIV-infected pregnant women delivering in this area yearly. Within the entire Hlabisa District, approximately 25-30% of women deliver at fixed clinics, 35-40% deliver at the Hlabisa District Hospital, and ~18% of women deliver at home. An additional 10-15% deliver at hospitals outside of the District or with GP’s in the District. Approximately 14% of first antenatal bookings occur at mobile clinics which work out of Hlabisa Hospital. The average gestational age of women attending for first antenatal booking is similar between primagravid (24.5 weeks) and parous women (25 weeks). The average age of all pregnant women booking in the district is 24.5 years, and 19.5 years for primagravids. GP’s in the district also offer a limited antenatal service to the community.

## 2.3 Socio-demographic profile

#### Mpukunyoni tribal area, Hlabisa district

This is a diverse area with altitudes ranging from 50-250m above sea level. There is one formal township at kwaMsane, under the jurisdiction of the Transitional Local Council, and the local tribal authorities govern the rest of the district. There is, accordingly, a broad socio-demographic profile. Residents of kwaMsane often have some category of professional living in the household (e.g. nurse, teacher, shop owner or government official), while in the rest of the district families are generally supported by a member working outside the district or supported by a government pension to older family members. A small number of businesses operate within Hlabisa. The provision of water and sanitation is similarly diverse. In kwaMsane most homes have piped water and water-born sewage while the rest of the district is largely dependent on wells and rivers and have only simple pit latrines or no formal sanitation facility.

## 2.4 Current infant feeding practices

In 1999, UNICEF accredited Hlabisa District hospital as ‘Baby Friendly’. As part of this all pregnant women and mothers attending the hospital and local clinics, both HIV-infected and uninfected, receive the same general infant feeding recommendation i.e. to EBF.

In September 1999, a cross-sectional survey of infant feeding practices was conducted in the Mpukunyoni tribal area. 447 mothers/caregivers of infants, aged 1 day to 12 months, attending local immunisation clinics were surveyed. Field workers asked about infant feeding practices in the preceding 48 hours and since birth and also information regarding home amenities. In addition, a longitudinal cohort of 130 mothers recruited antenatally tracked infant feeding practices within 96 hours of birth and at subsequent weekly home visits until 16 weeks. Mothers kept diaries to document days of EBF.

In the cross-sectional survey 87% of infants were receiving some breastmilk at the time of attendance for their immunisation. 60% of mothers reported giving fluids or feeds other than breastmilk by 3 weeks of age. 62% of respondents used lakes, rivers or ponds as their main water source. 31% had no toilet facility and 45% used wood as their main fuel source for boiling water. In the longitudinal study, 46% of infants received fluids or feeds in addition to breastmilk in the first 48 hours of life. Only 17% of infants were EBF to 2 weeks and 6% to 16 weeks of age.

# 3. Recruitment

## 3.1 Inclusion and exclusion criteria

#### 3.1.1 Inclusion criteria

1. All pregnant women attending antenatal clinics in the Mpukunyoni and Hlabisa Tribal Areas of Hlabisa District or at Kwadabeka clinic or St Mary’s hospital, Mariannhilll Hospital who offer consent to participate in the study.

#### 3.1.2 Exclusion criteria

1. Intention to leave Hlabisa District within 3 months of delivery.
2. Failure to give consent.
3. Less than 16 years of age.

#### 3.1.3 Multiple births

Twins and other multiple births will be eligible for study recruitment. For analysis of peripartum transmission data only the first infant of normal vaginal deliveries will be included. All infants born by caesarean section will be included. For analysis of postnatal transmission data all infants will be included as separate ‘units’ since feeding practices may differ significantly between infants.

#### 3.1.4 Women taking or starting concomitant anti-retrovirals

Women who are taking anti-retrovirals in the antenatal period or who start on treatment during the course of the study will be eligible for enrolment and may continue in the study. Details of treatment will be recorded and considered in the analysis.

#### 3.1.5 Women who have previously enrolled in the study

Women who previously enrolled in the study and whose child may or may not have participated e.g. previous child (+/-NVP) within study period, spontaneous abortion (no NVP) or stillbirth (+/-NVP) will still be eligible for enrolment.

#### 3.1.6 Women with clinical HIV/AIDS

Although it is unlikely that women with clinically advanced HIV/AIDS will become pregnant, they will still be eligible for inclusion in the study.

## 3.2 Voluntary counselling and testing

#### 3.2.1 General

In Hlabisa, the District PMTCT programme will offer voluntary counselling and testing for HIV at all clinics in Hlabisa district including those involved with the vertical transmission study. All HIV-infected women regardless of whether they enrol in the study will be offered nevirapine to take at the time of labour and for their child immediately post partum.

HIV counsellors from the Hlabisa District programme and the vertical transmission study team have been trained according to national guidelines and methods used at the government Aids Training and Information Centres (ATIC). In addition, all counsellors have been trained on the WHO courses: Breastfeeding counselling and HIV and Infant Feeding.

#### 3.2.2 Group education

All women attending the antenatal clinics for their first booking visit will receive, in groups of 10-20, general education about HIV, modes of MTCT, the benefits and risks of HIV testing, the benefits and risks of nevirapine. In clinics involved with the vertical transmission study there will also be a general introduction to the vertical transmission study.

#### 3.2.3 Pre-test counselling

HIV counsellors will involve women in a two-stage HIV pre-test counselling procedure after the group education. In the first stage, women will be counselled in small groups of 3-5 about personal risk assessment, types of HIV results, individual responses to a positive test, coping strategies and disclosure of HIV status and reducing transmission with an antenatal drug intervention. Women will be encouraged to bring partners along for testing. Women will be explicitly informed that a prophylactic regimen of NVP to the mother during labour and to the infant soon after birth will be available to those who test positive. In the second stage, either on the same day or at a later visit, women will be individually asked to indicate that they understand the decision and express their choice. Consent will be obtained at this time for HIV testing. Blood samples will be identified by a bar code number which will also be used to label her return appointment card. Women who want or need to discuss the matter with a family member or need more time to decide will be invited to return the next week. Samples will be sent to the Africa Centre laboratory in Durban for detection of anti-HIV antibody by ELISA. (See 9.2) Basic information re. parity, age, education and prior testing will be collected on all women to characterise ‘refusals’. Focus group discussions and exit interviews will be conducted to determine women’s understanding and the effectiveness of this approach.

#### 3.2.4 Post-test counselling

Women will be invited to return on specified dates after testing to receive their results from the HIV counsellor. Appointments will be given to permit adequate time for counselling. At these times women may also be invited to participate in the vertical transmission study and counselled on infant feeding options. The HIV counsellor will decide on the appropriateness and timing of these discussions depending on the woman’s response to learning her status. Counselling and testing will be offered to partners or siblings and the woman will be assisted to disclose her status to close family members or friends.

#### 3.2.5 HIV results - Mothers

Results will be delivered to the counsellor in the form of a Results List. This list will contain the barcode number used to identify the sample and the result. Counsellors will cross-reference the barcode number with the mother’s appointment card which will also be labelled with the barcode. In addition, at the time of testing, mothers will be asked to provide a key piece of information which will be recorded by the counsellor. At the time of post-test counselling the woman will be asked for her ID document and also this information to verify her identity. Women will be asked to sign that they have received their results and will be offered an official results slip. Results will not be routinely recorded on the antenatal clinic card.

Results will only be given out at clinics and HIV status will not be known to any member of the study team other than the study clinic team e.g. field-based teams. It is possible, however, that mothers will choose to disclose their status to field staff. Field staff i.e. breastfeeding counsellors and field monitors have been trained in ‘disclosure counselling’ for this possibility. If a woman does not return for results, she will not be traced at home nor offered results at home.

#### 3.2.6 Women testing positive for HIV

HIV-infected women will be counselled about their status and informed how to obtain NVP, when and how to take the intrapartum dose, and how to obtain NVP for their infant in the immediate post-partum period. (See 4.3) All HIV-infected women will then be screened for eligibility and invited to enrol into the study. All HIV-infected women, regardless of whether she chooses to enrol in the study or not, will then be counselled about infant feeding choices according to UNAIDS guidelines. (See 3.4)

.

#### 3.2.7 Women testing negative for HIV

Women who test negative for HIV will be advised to avoid unprotected intercourse and adopt other safer sex practices. Women will be advised to EBF their infants according to international recommendations. (See also 3.3.2)

#### 3.2.8 Women accepting HIV testing but declining to know status

A woman may indicate that she is willing to be tested (+/- participate in the study) but does not want to know her HIV status. This woman will be tested according to the protocol and the results retained by the HIV counsellor. If she later wants to know her HIV status then the counsellor will pre- and post-test counsel appropriately and offer nevirapine if indicated. The local district health service providers and national government have adopted the approach that nevirapine treatment should only be given after HIV testing.

#### 3.2.9 Women attending antenatal clinic and declaring to be HIV-infected

A woman who indicates that she is HIV-infected when she first attends the antenatal clinic will be asked to provide documentary evidence of her result before receiving NVP. The status of a woman who indicates that she is HIV-infected will be confirmed by a second sample before being enrolled into the study.

#### 3.2.10 Women booking at clinic after 36 weeks

Women booking after 36 weeks will be offered pre-test counselling and a rapid HIV test with results on the same day.

#### 3.2.11 Women arriving at the clinic in labour but HIV status unknown

Women arriving in the labour ward but whose HIV status is unknown will not be offered HIV testing before delivery. She will be offered testing after pre-test counselling in the early post-partum period and if HIV-infected the infant will be offered NVP.

## 3.3 Enrolment

#### 3.3.1 HIV-infected women

All HIV-infected women, regardless of their intended feeding practice, will be invited to participate in the study. Women will be screened according to the inclusion and exclusion criteria above. Eligible women will be invited to enter the study before the counsellor asks for an indication about the planned feeding practice. Some mothers may take several weeks to decide on the possible feeding options and may wish to discuss the issue with other important family members.

#### 3.3.2 HIV-uninfected women - random sample

A random selection of HIV-uninfected women will be invited to enrol in the same manner as above. Mothers identified for study inclusion will be identified to the HIV counsellor at the same time as obtaining HIV results.

This group is included to protect the confidentiality of HIV-infected mothers and to determine growth, development and morbidity patterns associated with infant feeding patterns and differences in EBF adherence rates.

#### 3.3.3 Women declining knowledge of HIV status

Women who indicate that they do not want to know their HIV status but who wish to participate in the study will be enrolled if HIV-infected or selected as part of the HIV-uninfected group. This approach has been adopted in other large community based studies, most notably the Zvitambo study in Harare.

#### 3.3.4 Study information and consent (Appendix A)

A written outline of the study in Zulu with details of home visiting and testing schedules will be given to each prospective study participant. Written consent will be requested for i). HIV testing, and ii). participation in the study. The mother will be offered a copy of the signed consent to keep as her own record. Mothers who cannot write will be asked to make a thumbprint on the consent form. One witness will be asked to verify all consents.

#### 3.3.5 Data collection

At enrolment women will be asked for detailed information to assist tracking and location of their home. This information will be returned to the centre and used to generate tracking sheets for later use. Counsellors will document the main reason for study refusal if a woman declines to enrol.

#### 3.3.6 First home visit

If a woman consents to enrol in the study, she will be visited at home by one of the field teams within one week. The purpose of this visit is to collect baseline demographic information and past medical/obstetric history and review the study routines with the women and any other family members she chooses to include. HIV status and intended feeding practice will not be discussed during this visit.

#### 3.3.6 Study number

A 6-digit study number will be assigned after the woman has agreed to participate. Mothers’ numbers will always conclude with ‘0’. The child will adopt the same first 5 digits of the mother’s study number but their study number will finish with 1 for singletons and 1, 2 or 3 respectively in the event of multiple births.

## 3.4 Counselling on infant feeding options (See UNAIDS recommendations - Appendix B)

#### 3.4.1 HIV-infected women – General description

Counselling will include a discussion of the relative risks of transmission of HIV from breastmilk and the risks of serious morbidity and mortality associated with formula feeds. The home and social circumstances of each woman e.g. support and attitudes of other family members will also be assessed. Women will be advised to avoid mixed feeding and to exclusively feed their children either breastmilk or formula feeds. Women who choose to breastfeed will be advised to EBF their child until about 6 months of age. HIV-infected mothers who choose to stop breastfeeding before then or who choose to introduce other foods or fluids before then, will be advised that mixed feeding is the least safe option and, as far as we know at present, EBF or exclusive replacement feeds are more likely to avoid vertical transmission of HIV. Women will be supported to continue EBF or to safely replace all breastmilk.

When the child is 4-6 (18-26 weeks) months of age, HIV-infected mothers will again be counselled on feeding practices but for the child’s second 6 months of life. Mothers will be advised that all children need to receive complementary feeds but that special consideration is needed to determine what may be best for her child. These factors will include the HIV status of her child at that time, home circumstances e.g. water and sanitation and other risk factors that may be associated with replacement feeds e.g. sustainability of breastmilk alternatives. To assist this exercise, children of HIV-infected mothers will be tested for HIV using real-time molecular PCR methods at ~22 weeks of age.

#### 3.4.2 HIV-infected and uninfected women choosing to breastfeed

HIV-infected and uninfected women who wish to breastfeed will be given support and encouragement to EBF. This service will be provided to women who do not enrol in the study by District health staff. HIV-infected and uninfected women who do enrol into the study will receive breastfeeding counselling and support at their homes for the first 6 months of life and at clinics thereafter. The breastfeeding counselling message and support will be independent of HIV status and field-based breastfeeding counsellors will be blinded to the HIV status of all participants.

HIV-infected mothers who have been breastfeeding and whose children are uninfected will be advised to replace all breastfeeding at ~6 months and offer a safe and adequate replacement milk in addition to complementary feeds. Advice will be offered regarding culturally acceptable, locally available, affordable and nutritionally adequate complementary feeds and breast milk substitutes such as fermented cow’s milk (maas) or formula milk. Haemoglobin will be checked monthly on infants aged between 6 and 9 months and 3 monthly between 10 and 24 months. Iron supplements will be provided to those children whose haemoglobin falls below 8 gm/dL.

HIV-infected mothers with children who are also infected by 4-6 months of age, and all HIV-uninfected mothers will be advised to continue breastfeeding until their children are 18-24 months of age in addition to complementary feeds. Haemoglobin will be checked as above and iron dispensed as described.

#### 3.4.3 HIV-infected women choosing to exclusively formula feed

HIV-infected mothers who opt to formula feed will be supported in their choice by HIV counsellors based at clinics. For this choice to be considered safe and reasonable, it requires mothers to have access to clean water and an uninterrupted supply of formula milk for the first 6-9 months of age and to be given appropriate advice on safe formula feeding. The HIV counsellors have been trained in safe preparation and use of formula feeds and will be able to demonstrate best practices to these mothers. Advice on the addition of complementary feeds and continuing replacement feeds will be given when the child is 4-6 months old.

# 4. Antenatal care

## 4.1 Initial history and examination

Women attend antenatal clinics according to the routine schedule i.e. 4 weekly visits until 36 weeks, then 38 weeks and weekly thereafter until delivery. All women consenting to the study will have a routine antenatal history and physical examination. This will include verifying the date of last menstrual period and intentions regarding site of delivery, infant feeding practice and plan to return to work or school. A detailed description will be taken of home location for future visits. The District health nurse will perform a standard antenatal examination including weight check, measurement of blood pressure, listening to the fetal heart and urinalysis for protein and sugar. At subsequent visits all women will receive general information about breastfeeding from clinic staff.

## 4.2 Gestational age assessment

The gestational age of infant and estimated date of delivery will be determined by the date of last menstrual period. If the woman is uncertain about her dates, then the dates will be based on a fundal height assessment on clinical examination. If the woman attends for an ultrasound assessment before 20 weeks then her dates may be revised based on fetal bi-parietal diameter measurements.

## 4.3 Antiretroviral intervention (Nevirapine)

#### 4.3.1 Dispensing of maternal dose of nevirapine

District health staff will give HIV-infected women, at ~30-34 weeks gestation, a NVP pack, which will include a 200mg tablet of nevirapine and instructions for use at the time of labour/delivery. The mother will be advised to take the medicine once it is certain that labour is established, whether she is at home or in a facility. She will also be advised that the child similarly needs a dose (see dosing schedule below), but this will be dispensed at the clinic or hospital where the baby is born. Women will be advised that in the event of delivering at home, she should bring the baby to the clinic as soon after delivery as possible.

#### 4.3.2 False labour

In the event of the mother taking the NVP dose but labour failing to progress to delivery, a repeat dose will be dispensed and the mother will be instructed to repeat the dose during labour if delivery does not occur within 24 hours.

#### 4.3.3 Infant postpartum dose

District health staff assisted by clinic/hospital study teams will dispense NVP to newborn infants within 72 hours of delivery. This will generally be given at the same time as the BCG and first oral polio vaccination. All infants >2kg will be given 6 mg (0.6ml) PO. Infants <2kg will be given NVP 2mg/kg. In the event that a mother does not take her NVP or if she takes the tablet less than one hour before delivery then NVP will be given as soon after delivery as possible. If the mother takes her dose within 2 hours of delivery the infant will be given a first dose of NVP as soon after birth and a second dose 24-48 hours later. In the event that a mother delivers at home and the infant is not brought to the clinic immediately then NVP will still be given to infants brought up to 7 days of age.

#### 4.3.4 Dose and supplier information

- Dose, mother and infant Mother – 200mg orally at onset of labour

Infant >2kg - 6mg (0.6ml) within 72 hours of delivery

Infants <2kg – 2mg/kg within 72 hours of delivery

- Formulation Mother – single 200mg tablet

Infant - Solution

- Supplier Boehringer Ingelheim Pharmaceuticals

#### 4.3.5 Concomitant medicines including anti-retrovirals

Women taking concomitant medicines including anti-retrovirals from a source outside the study will be given NVP and advised to follow the same protocol as above. Details of such medication will be recorded.

#### 4.3.6 Safety and adverse events

NVP is registered in South Africa for use to reduce mother to child transmission of HIV. Consequently, consent will not be requested, nor will any specific screening be implemented before dispensing. Any unusual symptom reported by the mother or clinical sign detected by the study clinician within two weeks after treatment will be recorded and assessed as a possible adverse event related to NVP treatment. Adverse event will be reported to the South African Medicines Control Council.

## 4.4 Blood samples

All women booking at clinics are tested for syphilis and haemoglobin at their initial visit. This same sample will be tested for HIV in women who consent to testing.

HIV-infected women who consent to the study will have a second blood sample taken at the enrolment visit, which will be tested for HIV viral load and CD4 count.

## 4.5 Folate and Iron

All pregnant women receive folate 5mg daily and ferrous sulphate from the first antenatal booking visit until delivery as part of routine antenatal care in Hlabisa district. If haemoglobin levels fall below 9 gm/dL at any time the woman will be referred to the medical officer.

## 4.6 Cotrimoxazole

HIV-infected women who have CD4 counts less than 200/mm^3^ or who have symptomatic HIV/AIDS will receive cotrimoxazole (trimethroprim 80mg / sulfamethoxazole 400mg – 2 tablets daily) through the routine mechanisms at the district clinics. This is consistetnt with SA National policy. (See 8.3.5. regarding prophylaxis to infected infants)

## 4.7 Home visits

Trained breastfeeding counsellors will visit women at home who enrol into the study and who indicate to the HIV counsellor that they intend to breastfeed. Visits will start at 36 weeks and continue weekly until delivery of the child. At these visits breastfeeding counsellors will discuss techniques for optimal breastfeeding and pitfalls that may occur during the first 1-2 weeks of life. Breastfeeding counsellors will aim to identify, with the woman, some person at home who will support and facilitate her breastfeeding. See below.

# 5. Delivery

## 5.1 Obstetric practices

There are only two facilities in Hlabisa district which offer a 24 hour, 7 day per week, delivery services. The District health services have implemented obstetric practices to minimise the risk of mother to child transmission of HIV. These include:

- Avoidance of routine episiotomies
- Avoidance of artificial rupture of membranes, and
- Minimal instrumentation

## 5.2 Data collection

Data will routinely be collected following delivery of the infant. This will include information on

- Treatment for STD’s during pregnancy
- Duration of labour and ruptured membranes
- Episiotomy or tear requiring stitches
- Mode of delivery
- Reason for Caesarean section if appropriate
- Delivery outcome (live birth, stillbirth or spontaneous abortion)
- Apgar count
- Early neonatal outcome
- Birth weight, length and head circumference
- Interval (hours) between mother taking NVP and delivery
- Time when NVP given to infant

## 5.3 Vitamin A supplements at delivery

All mothers routinely receive vitamin A 200,000IU after delivery through the routine district health services.

# 6. Post-natal home visits - Breastfeeding counselling and support strategy (BCSS)

## 6.1 General description

A comprehensive breastfeeding counselling and support strategy has been developed. This has been based on a recently completed ethnographic survey of attitudes toward infant feeding choices and HIV, and a detailed description of feeding practices in Hlabisa district. The strategy has been piloted to determine its acceptability and effectiveness.

## 6.2 Breastfeeding support team

Participating women will be supported in their choice to breastfeed by a community-based team of breastfeeding counsellors. These breastfeeding counsellors have been trained in the WHO Breastfeeding Counselling Course. A supporting field manual based on this course has been devised for on-going training and to ensure a consistent approach to all advice. In addition, all health staff at clinics in the Hlabisa area are being trained in breastfeeding management as part of the UNICEF Baby Friendly and Integrated Management of Childhood Illnesses initiatives. Breastfeeding counsellors have been trained specifically to address the problems of thirst, fluid management of diarrhoea, constipation, breast care and perceived milk insufficiency syndrome and how to provide psychological support to the mother.

## 6.3 BCSS schedule and procedures

#### 6.3.1 Antenatal schedule – See 4.6

All women enrolled in the study who choose to breastfeed, regardless of HIV status will be seen weekly by a breastfeeding counsellor from 36 weeks onwards to discuss concerns and the feasibility of EBF until 6 months. (see 4.6) During these visits which will occur at home, members of the family will be encouraged to support the mother in her decision to EBF. The woman will be asked to identify a ‘home support’ - another family/household member who can assist at home and provide support over the following months. At subsequent antenatal visits, problems and concerns specifically relating to initiating breastfeeding and the first 1-2 weeks postpartum will be addressed with the woman and home support.

#### 6.3.2 Home support (HS)

This person would ideally be chosen by the mother and would be living in the same household and had previously successfully breastfed her own infant. It may however be a supportive sibling without a child of his or her own. In exceptional circumstances, the father of the child may be the HS. This person will be trained in the technical aspects of breastfeeding and also sensitised to the cultural and social importance of breastfeeding. This person should receive all the same information about EBF and infant health, as does the mother. The field monitoring teams will ask the HS about the mother’s infant feeding practices.

#### 6.3.3 Post-natal strategy

After delivery, the breastfeeding support team will visit the mother and HS at home within 96 hours of delivery. Mothers will be visited twice weekly for the first 2 weeks of life and on alternate weeks thereafter. The breastfeeding counsellor may, at her own discretion, make additional visits according to the age of the child or condition of the mother. The strategy is intended to provide support and encouragement to mothers at regular intervals and to focus on the most vulnerable times and threats to EBF. Mobile teams will be able to respond to urgent needs of a mother or HS.

#### 6.3.4 Formula feeding

Mothers who choose to formula feed from birth will be given advice on how to safely prepare and store feeds by the clinic-based HIV counsellor. Neither formula milk nor bottles and teats will be given out as part of the study. Monitoring of feeding practices will be undertaken within the home to ensure the safest possible techniques. The use of cup and spoons will be encouraged as an alternative. These mothers and children will be followed until 24 months of age but will not be included in the sample size calculations. Home-based support will be offered by study nurses at the clinic. If accepted, study nurses will advise field staff to start home visits. Visits will be conducted every 2 weeks, or more or less frequently depending on the mother’s preference.

#### 6.3.5 Mothers returning to work or school

Mothers who plan to return to work or school will be offered the use of breast-pumps to facilitate expression.

#### 6.3.6 Breast health and EBF adherence

Field breastfeeding counsellors will obtain specific data regarding breast health which may be relevant to HIV transmission e.g. cracked or bleeding nipples, mastitis or breast engorgement. This information is also required by the breastfeeding counsellor to effectively counsel. Breastfeeding counsellors will not know the HIV status of mothers and advice given will be independent of HIV status. Serious problems of breast health e.g. mastitis or breast abscess will be immediately referred to the clinic for appropriate management.

#### 6.3.7 Breast thrush

Breastfeeding counsellors will provide nystatin cream to mothers and suspension for children if she diagnoses breast thrush.

6.3.8 Cessation of breastfeeding and complementary feeds

Women will be advised and supported to continue EBF up to 26 weeks of age. However, children will be tested for HIV status at 22 weeks of age and the result made available to the study HIV counsellor based at each clinic. These results will be held until the mother returns with the infant at 24-26 weeks of age when the result will assist counselling on subsequent feeding recommendations.

- Mothers of children who are already infected will be advised to continue breastfeeding until the child is at least 2 years of age.
- Mothers of children who are uninfected will be encouraged to stop breastfeeding over the shortest possible time. Advice will be offered regarding culturally acceptable, locally available, affordable and nutritionally adequate complementary feeds and breast milk substitutes such as fermented cow’s milk (maas) or formula milk. As the field breastfeeding counsellors are unaware of the HIV status of the mother and child, these mothers will be asked to attend the clinic for continuing breastfeeding and complementary feeds advice. Haemoglobin will be checked on infants monthly and iron supplements will be provided for those children whose haemoglobin falls below 8 gm/dL.
- Women who are HIV-uninfected will be advised to continue breastfeeding until 2 years of age and introduce a balanced diet of complementary feeds from 26 weeks of age.
- Breastfeeding counsellors will routinely discuss locally available and affordable complementary feeds with all mothers from 18 weeks onwards.
- If a field monitoring team establishes that a mother has opted to change to MBF before 26 weeks of age, the mother will be asked to visit the study team based at the clinic, who will have access to HIV status and will advise accordingly.

#### 6.3.9 Modification of BCSS

BCSS may be modified as experience grows in the research team and the study population. The strategy will be periodically reviewed and modified to achieve the maximum number of women EBF at 6 months. Changes to the breastfeeding support intervention do not alter the primary transmission endpoints, but only the rate at which the study achieves the number of evaluable subjects who EBF at a given time point.

# 7. Post-natal home visits – Field monitoring

## 7.1 General description

All mothers and infants recruited into the study, regardless of whether they are HIV-infected or uninfected, or whether they breastfeed or formula feed will be visited at their homes to collect data on feeding practices and morbidity. Specifically information will be collected on diarrhoea and respiratory morbidity and concurrent medicines being taken. A data collection tool has been developed and piloted in the past year to record information on feeding practices which can be organised within the database either as a continuous variable or as single event.

## 7.2 Field monitoring team

An independent team of field monitors will collect feeding and morbidity data. This approach is aimed at reducing any bias in data reporting that may occur if the breastfeeding counsellors were to collect data as well as counsel on feeding practice.

## 7.3 Field monitors schedule and procedures

#### 7.3.1 Weekly visits

Field monitors will visit homes within one week of delivery and continue weekly visits until 6 months of age or until all breastfeeding ceases, whichever is later, to document infant feeding practices.

##### 7.3.2 Feeding diaries

Mothers will be asked to keep diaries of food intake for the preceding week. The field monitor will check this at every visit. If the mother is giving any fluids/foods other than breastmilk, then the nature, frequency and volume of these fluids/feeds will be recorded. This method has been piloted and found to be reliable and a useful addition to field methodology.

#### 7.3.3 Data collection

Feeding practices in the week preceding visits will be documented using a standard process. Firstly, the inclusion of any milk, fluid or food other than breast milk will be established for each day since the last visit. Secondly, an average daily record of the frequency and volume of milks, fluids and feeds other than breast milk will be documented for the preceding week.

Data will be corroborated by inspection of the diaries, interviews with the HS, and by observation of the home environment e.g. presence of bottles or infant feeds in the homestead.

Diarrhoea (maternal definition) and other specific morbidity will be recorded for each day since the last monitoring visit. The number of stools on the worst day of illness will be recorded.

#### 7.3.4 Data validation

Data from weekly interviews will be validated by a system of repeat interviews. Every 40^th^ weekly feeding assessment will be repeated the following day by a field supervisor. The weekly questionnaire will be asked of the same informant as the previous day, and the fieldwork manager will reconcile discrepancies between responses. ‘Query’ sheets will be sent to the field monitor who conducted the first interview, to outline the discrepancies and results.

#### 7.3.5 Health education and advice

The field monitoring teams will have no responsibility for providing health counselling or advice about breastfeeding management. If family members at home seek advice, the monitoring team will alert their field supervisor who will communicate the need to the breastfeeding support field supervisor, who will mobilise the appropriate response. All field workers will be trained in basic health education and first aid to respond appropriately to emergencies in the field.

# 8. Post-natal clinic visits

## 8.1 General description

Women and children will attend clinics for routine antenatal care and immunisation as normal. The study schedule has been organised to make use of these visits as much as possible. Study nurses will work closely with the District staff to provide a seamless service and one that benefits the community, health services and research team. Routine functions that overlap e.g. routine weighing and measuring may be performed by the study team and recorded in infants Road to Health Cards as a way of assisting local health staff.

## 8.2 Study clinic team

The research study team will consist of an HIV counsellor, study nurse and a clinic assistant. The role of the counsellor has been described above. The nurse will see women and infants at each clinic visit and will assess growth and development and take blood samples according the schedule below. The nurse will also accept referrals from field staff in the event of serious breast health difficulties and for detailed counselling on infant feeding and nutrition. Clinic assistants will assist with routine anthropometry, blood sampling and labelling of dried blood spots and breastmilk samples. They will also give group education talks on HIV (as above) and also on breastfeeding. This staff have all been trained on breastfeeding counselling.

## 8.3 Clinic visit schedule, care and procedures

#### 8.3.1 Times of visits

Mothers and infants will be seen at 6, 10 and 14 weeks to coincide with routine immunisations, and then at 18, 22 and 26 weeks of age. After 26 weeks (6 months) mothers and infants will be seen at 7, 8, and 9 months to assess growth, adequacy of nutritional intake after the introduction of complementary feeds and to determine haemoglobin levels. Mother and infants will be seen three-monthly thereafter to monitor growth and collection of HIV diagnostic blood samples. See table 1, page 9.

#### 8.3.2 Clinical assessments

Mothers will be asked about feeding practices in the preceding week, infant morbidity episodes and breast health i.e. cracked and/or bleeding nipples, mastitis, abscesses etc. Infants will routinely be measured for weight, length and head circumference, and examined by the research nurse for clinical signs suggestive of HIV infection i.e. generalised lymphadenopathy and recurrent oral candidiasis. Between 6-9 months particular attention will be taken to assess growth and the adequacy of nutritional intake at the time when complementary feeds are being introduced. The child’s Road to Health Card will be checked on each occasion to ensure that all immunisations are up to date.

#### 8.3.3 Growth faltering

If a child is found to be growth faltering at any stage, study nurses will first assess the child clinically to identify an organic cause for the problem and take a detailed feeding history. Study nurses will work in cooperation with the local health staff and the project paediatrician to determine if there is an underlying condition that can be treated or if the child has HIV infection. If the child is growth faltering because of inadequate food intake mothers may be referred to the District Protein Energy Malnutrition (PEM) programme. This programme is intended to assist mothers who have children with PEM and provides beans and dried milk powder for the mother and child.

#### 8.3.4 Biological samples - see Table 2 below

Blood will be collected from all women at their first antenatal booking. This will be tested to determine HIV status and, in those who are infected, viral load and CD4 counts will be established on the subsequent sample. Samples of women who were HIV-infected at booking will be tested postpartum at 6 weeks for NVP resistance and at 22 weeks for HIV viral load and CD4 count. Samples of women who were HIV-uninfected at booking will be re-tested for HIV status at 6 and 22 weeks postpartum. The incidence rate in South Africa and particularly KwaZulu Natal may be >10% and it is possible that a number of HIV-uninfected women will become infected during pregnancy.

Breastmilk samples from each breast (20mls) will be requested from all mothers within 72 hours of birth and at each routine clinic attendance i.e. 6, 10, 14, 18, 22 and 26 weeks. Three 50µl spots will be placed on filter paper and stored. The remainder will be split into three aliquots and stored at –70^o^c. Breastmilk will be analysed for viral load, Na^+^:K^+^ ratio and other transmission factors.

Blood samples will be obtained from infants by finger or heel prick and stored on filter paper i.e. dried blood spot (DBS). See section 9 for rationale of DBS and laboratory procedures. DBS samples from infants born to HIV-infected mothers will be collected at birth, 6, 10, 14, 18, and 22 weeks of age. A venous blood sample will be obtained at 26 weeks. The 6 and 22 weeks samples will be tested real time and the results will be available within two weeks in order to direct decisions on providing cotrimoxazole and feeding practices in the second 6 months of life. Other samples will be collected only if the real-time PCR test of previous sample at 6 and 22 weeks was negative for HIV. Samples will be stored and analysed later to determine the timing of acquisition of infection by infants who ultimately become HIV-infected. See section 9.

Table 2. Blood and breastmilk sampling schedule

|  | 1^st^ A/N visit | 2^nd^ A/N visit | Birth <72 hrs | Wk  6 | Wk  10 | Wk  14 | Wk  18 | Wk  22 | Wk  26 | Mth  7 | Mth  8 | Mth  9 | Mth  12 | Mth  15 | Mth  18 | Mth  21 | Mth  24 |
| --- | --- | --- | --- | --- | --- | --- | --- | --- | --- | --- | --- | --- | --- | --- | --- | --- | --- |
| **Women (All)**  Hb  Syphilis  HIV ELISA | X^†^ |  |  |  |  |  |  |  |  |  |  |  |  |  |  |  |  |
| **Women**  **(HIV-infected)** |  |  |  |  |  |  |  |  |  |  |  |  |  |  |  |  |  |
| Viral load and CD4 |  | X |  |  |  |  |  | X |  |  |  |  |  |  |  |  |  |
| NVP resistance |  |  |  | X |  |  |  |  |  |  |  |  |  |  |  |  |  |
| Haemoglobin |  |  |  |  |  |  |  |  |  |  |  |  |  |  |  |  |  |
| Breastmilk for viral load and Na:K ratio |  |  | X | X | X | X | X | X | X |  |  |  |  |  |  |  |  |
| **Women**  **(HIV-uninfected)** |  |  |  |  |  |  |  |  |  |  |  |  |  |  |  |  |  |
| HIV ELISA |  |  |  | X |  |  |  | X |  |  |  |  |  |  |  |  |  |
| Breastmilk for Na:K ratio  ± viral load |  |  | X | X | X | X | X | X | X |  |  |  |  |  |  |  |  |
| **Infants**  **(HIV+ mothers)** |  |  |  |  |  |  |  |  |  |  |  |  |  |  |  |  |  |
| RNA PCR* |  |  | X | X^†^ | X | X | X | X^†^ | X^‡^ |  |  | X | X |  |  |  |  |
| HIV ELISA |  |  |  |  |  |  |  |  |  |  |  |  |  | X | X | X | X |
| Haemoglobin |  |  |  |  |  |  |  |  | X | X | X | X | X | X | X | X | X |
| **Infants**  **(HIV- mothers)** |  |  |  |  |  |  |  |  |  |  |  |  |  |  |  |  |  |
| Haemoglobin |  |  |  |  |  |  |  |  | X | X | X | X | X | X | X | X | X |
| DBS for possible HIV testing |  |  | X | X | X | X | X | X | X |  |  | X | X | X | X | X | X |

X = time samples taken

* Samples collected only if real-time test of previous sample at 6 and 22 weeks was negative for HIV

^†^ Sample processed real-time

^‡^ Venous blood sample

#### 8.3.5 Cotrimoxazole prophylaxis

Cotrimoxazole prophylaxis will be provided to all children who are HIV-infected when tested at 6 weeks or 22 weeks of age and will continue until 12 months of age. This is in keeping with the Natal Provincial clinical care guidelines for children with HIV.

#### 8.3.6 Vitamin A

National and WHO guidelines recommend that all mothers will receive vitamin A (200,000 IU) orally after birth. This has not, however, been implemented as yet by the District health services. If implemented this information will be recorded and included in the data analysis.

#### 8.3.7 Data collection

Routine anthropometry, clinical status and morbidity information from unscheduled clinic visits will be recorded at each clinic visit. Reasons for change of feeding practice will be documented to inform analysis of data and possible reverse causality. Information will be recorded in carbonised study books to allow immediate data capture but also for a record to remain at the clinic for reference.

#### 8.3.8 Failed clinic attendance

If a mother and infant fail to attend for a clinic appointment, the field monitoring team will visit the mother at home and assess the reason for non-attendance. An appointment will be given for an early clinic visit.

#### 8.3.9 Unscheduled clinic attendance

District clinic nurses will see infants who are brought to clinics for unscheduled visits, and morbidity events documented on the Road to Health Card. These events will be documented by the study clinic team at the next scheduled clinic visit.

# 9. Sampling methods, Diagnostic algorithms and Laboratory methods

## 9.1 Sample collection and handling

#### 9.1.1 Venous blood samples

Venous blood samples (3-5ml) will be collected using the vacutainer system. In addition, if women agree to HIV testing then blood drawn will be used to perform rapid HIV tests as described in the South African national PMTCT protocol.

Two samples will be obtained on each occasion. The first will be placed in a plain tube (clotted sample) and the second will be placed into an EDTA containing vacutainer tube which will be inverted 7 times immediately on collection. Samples will be bar-coded and placed in individual sealing plastic bags. Samples will be put in cold storage and transported in refrigerated containers to the Africa Centre virology laboratory in Durban.

In the laboratory, three 50 µl samples of whole blood will be spotted onto filter papers, air dried and stored for later processing. Clotted blood will be centrifuged at 800 X g and two 50 µl aliquots of serum spotted onto filter paper or stored in 1 ml frozen aliquots at -80 C.

#### 9.1.2 Dried blood spots

Dried blood spots will be obtained by finger or heel pricks using a single-use, automated lancet with retractable blade (Softclix Pro Lancet, Boehringer Mannheim) and collected onto filter paper (Schleicher and Schuell, Keene, NH) by clinic-based research staff. Samples will be labelled with bar codes and placed in individual envelopes. Envelopes will be collected from the clinics each day and brought back to the Africa Centre. Filter papers and envelopes will be hung out until completely dry (usually 4-6 hours). Filter papers and envelopes will be placed inside a sealing plastic bag with desiccant granules. An independent register of samples will be maintained in the Africa Centre. Samples will be transferred for storage at –20ºC and analysis at the Africa Centre virology laboratory in Durban.

#### 9.1.3 Rationale for using dried blood spot (DBS) specimens

The collection of large numbers of blood samples for epidemiological and clinical studies is expensive for many reasons. In the field, obtaining venous blood can be difficult and time consuming, especially from infants and small children. This may reduce participation, requiring the recruitment of larger populations and raising concerns about enrolment bias. Safety issues must also be considered. Once obtained, venous samples require appropriate on-site processing for serum, plasma or cells. When large numbers of samples are collected, field conditions may limit the prompt handling and refrigeration of samples, leading to haemolysis and possible bacterial contamination. In addition, shipping of liquid samples requires maintenance of the cold-chain, adding weight and volume to the shipment and risking delays en route that could ruin the samples. The collection of dried specimens (finger- or heel-prick) on filter paper provides a powerful and practical solution to many of these problems. Blood sampling by lancet prick is familiar to populations in developing countries since blood slides are commonly used for malaria smears and microhaematocrit tubes are used for assessing anaemia. Collection requires little formal training, the supplies are inexpensive and the samples are easily transported because they are safe, lightweight and biologically stable. DBS testing has proven particularly valuable for the prompt and definitive diagnosis of HIV at birth, and to facilitate routine monitoring of neonatal viral loads to ensure timely intervention and appropriate therapeutic protocols. A wide range of serological (IgG and IgA ELISA, min-Western blot, p24 antigen capture), immunological (CD4 and CD8 ELISA) and virological testing (DNA PCR, RNA viral load, resistance genotyping) is possible. This approach provides access to the sophisticated molecular technologies that were previously available only to those residing near large urban technology centres.

## 9.2 Determination of HIV status and CD4 count

#### 9.2.1 HIV status - Women

HIV status will first be determined by the use of two rapid HIV tests as per the South African PMTCT protocol. Samples testing positive will be confirmed with a further Rapid test as per the South African PMTCT protocol and also according to the protocol in the following paragraphs.

Mothers will be considered HIV-infected if they have a positive result for anti-HIV-antibodies by enzyme-linked immunosorbent assay (Vironostika, Omnimed) which is confirmed by a second ELISA (GAC, Abbot) or Western blot.

Mothers who present late in pregnancy, in labour or at mobile clinics will have an initial sample tested using a rapid on-site test kit. Samples from mothers who are positive by this test will be forwarded to Durban for confirmation by standard tests. The woman will be considered infected on the basis of the rapid test and will be offered NVP and enrolment into the main study if appropriate. Samples from women who are negative on this test will not be sent for further analysis.

#### 9.2.2 HIV status - Infants

Testing for HIV status at 6 and 22 weeks will be performed on blood-spot samples using the Organon Teknika quantitative HIV RNA assay. This assay has a sensitivity of 1600 copies per 50 μl sample. This is equivalent to 80 copies per ml. The following algorithm will be followed to determine HIV status:

- *Samples collected at 6 and 22 weeks will be tested real time with the Organon Teknika PCR assay.*
- *If HIV RNA not detectable (<80 copies/ml), then the assessment is NEGATIVE ASSAY (NOT HIV-INFECTED)*
- *If 80-10,000 copies/ml, an additional sample will be obtained from the child >72 hours after the initial sample. If this second sample is >10,000 copies/ml the child will be classified as HIV-infected. If < 10,000 copies/ml the child will be classified as HIV-uninfected.*
- *If the initial sample is >10,000 copies/ml a second sample will be obtained. If there are >10,000 copies/ml in the second sample then the child will be classified as HIV-infected, and the parents or guardians informed accordingly. In the rare event that the second sample has a concentration < 10,000 copies/ml, a third sample will be obtained. Classification will then be based on the results of this third sample (< 10,000 copies/ml, HIV-uninfected; > 10,000 copies/ml, HIV-infected).*

This algorithm for diagnosis is consistent with that recommended by the Ghent International Working Group on Mother-To-Child Transmission of HIV. Because we will also obtain samples for HIV RNA testing at 9, 12, 15, 18 and 24 months the validity of this working definition of status can be confirmed. In limited studies to date we have not found false positives with the Organon Teknika assay in children.

Samples obtained at 15 months or later will be tested using the same method as above for women.

#### 9.2.3 CD4 counts – Women

CD4 cell counts will be measured on 1 ml of heparinized blood using a fluorescent activated cell sorter.

# 10. statistical analyses and Sample size calculation

## 10.1 General considerations

The analysis will aim to clarify mechanisms and risk factors by which HIV infects the breastfed young child in the post-natal period. It is currently unclear whether EBF, if it does reduce transmission, mediates its effect through the composition of breastmilk e.g. by reducing milk stasis, mastitis and viral load; by protecting the integrity of the infant gut mucosa by the avoidance of contaminated water or foreign antigens; promoting earlier gut maturation; by regulating immune responses; or a combination of the above. Classifying breastfeeding into a dichotomous variable (EBF and MBF) may obscure associations of interest. It is plausible that any transmission effect is based on a continuum of feeding practices rather than an all or nothing effect. The analysis plan therefore needs to respond to the relative success or failure of the intervention and to the possible pathophysiology of HIV transmission.

The primary objective of the study is to determine the HIV infection rates of infants at 6 and 22 weeks of age and to determine the effect of feeding practices on these rates. HIV tests performed on samples collected within 72 hours of birth inform about transmission of HIV that occurs *in utero*; tests on samples collected at 6 weeks of age inform about transmission that occurs during delivery. HIV tests performed on samples collected thereafter, inform about transmission that occurs due to the mode of infant feeding practiced up until ~4 weeks before the sample was collected. Hence samples collected at 22 weeks inform about transmission that has occurred in children who have been breastfeeding, exclusively or otherwise, at least until ~18-20 weeks. For clarity, reference to transmission rates in this section will include the relevant duration of feeding practice in parentheses.

## 10.2 Assumptions re. transmission and feeding patterns for sample size calculation

1. HIV infection rates at 6 weeks represent all *in utero* and intra-partum transmission, in addition to some possible early breastmilk transmission.
2. HIV infection rates of infants at 6 weeks of age born to HIV-infected mothers and who both receive NVP will be determined and is expected to be ~11%.
3. Transmission attributable to EBF will be inferred by any transmission that occurs after 6 weeks in a compliant EBF population.
4. The infant blood sample taken 4 weeks after the cessation of EBF will be the end-point for EBF-associated transmission.
5. Hence, for example, a blood sample taken from EBF infants at 22 weeks will reflect the transmission that has occurred through 18 weeks of EBF.
6. An HIV infection rate in EBF infants at 22 weeks of age more than 5% greater than the HIV infection rate at 6 weeks of age will be taken as an arbitrary but significant increment.
7. ~5% of women in the District will choose to EFF their infants from birth.

## 10.3 Study endpoints

#### 10.3.1 Primary endpoints

1. HIV infection rates of infants at 6 weeks of age who are EBF, MBF or EFF as determined by HIV RNA PCR analysis of a blood sample at that time.
2. HIV infection rates of infants at 22 weeks of age representing the effect of 18 weeks feeding practice as determined by HIV RNA PCR analysis of a blood sample at 22 weeks.
3. Infant survival rates at 24 months of age.

#### 10.3.2 Secondary endpoints

1. HIV infection rates of infants within 72 hours of birth as determined by HIV RNA PCR analysis of a blood sample obtained in that time period.
2. Difference between intrauterine and intrapartum HIV infection rates in women and neonates receiving NVP as determined by HIV RNA PCR analysis of blood samples collected within 72 hours of birth and at 6 weeks.
3. HIV infection rates of infants at various time points between 6 and 26 weeks, in relation to the type and duration of infant feeding practices i.e. EBF, MBF and EFF (+/- an initial period of EBF).
4. HIV infection rates of infants 4 weeks after cessation of EBF as determined by HIV RNA PCR analysis of blood samples collected at that time.
5. Prevalent days of diarrhoea.
6. Prevalent days of respiratory distress.
7. Changes in weight, length and head circumference.
8. Maternal morbidity.
9. Percentage of women who adhere to EBF.

## 10.4 Statistical analyses

10.4.1 General description

Assuming that a sufficiently large cohort of women who EBF until at least 22 weeks of age will be achieved, transmission rates in the children of these women at 6 and 22 weeks will be reported with reasonable confidence intervals. Multiple logistic regression analyses will be performed to ascertain the importance of time and quantity/volume dependent variables, calculating adjusted odds ratios and their confidence intervals. Options to be considered in these analyses include early and late addition of non-breastmilk feeds and the relative contribution of non-breastmilk feeds to the overall nutritional intake. The influence of both maternal and infant morbidity will be assessed in relation to feeding practice and transmission. Finally, Kaplan-Meier product-limit analysis (conventional survival analyses) will be performed to determine the cumulative age-adjusted incidence of vertical transmission for EBF. Time-dependent Cox regression analysis will take into account the dynamics of exposure and acquisition of infection over the first 26 weeks of life.

If the number of women who EBF until 22 weeks of age is not sufficiently large, the analysis plan will be adjusted to concentrate on:

1. Comparison of the HIV infection rate at 22 weeks of age by the feeding practice at the 4, 6, 8, 10, 14 and 18 weeks visit (i.e. cross-sectional approach)
2. Comparison of the HIV infection rate at 22 weeks of age by the maintained feeding practice until 4, 6, 8, 10, 14 and 18 weeks of age.
3. Refining the definition of EBF. The field monitors will collect data regarding the frequency and volumes of feeds and fluids that are given other than breast milk. With this information it will be possible to apply different feeding categories to the children in the study and investigate if there are threshold effects for transmission.
4. Cumulative incidence of vertical transmission for EBF, MBF and EFF groups using post-hoc definitions of breastfeeding categories.

After the first year of enrolment an initial estimation of adherence to EBF will be conducted. This estimate will inform the likely size of an EBF cohort, which will enable us to more accurately plan the analyses. Given that this is an implementation study, HIV infection rates will be reported according to i) the entire cohort of mothers who are exposed to the BCSS intervention i.e. similar to an intention to treat analysis, and ii) the feeding groups which emerge in the course of the study. It is worth emphasizing that feeding and transmission data on all mother and infants will be collected until 26 weeks regardless of whether they remain EBF, EFF or not. If the child is uninfected at 22 weeks and still breastfed, transmission data will be collected until 4 weeks after the mother stops breastfeeding.

#### 10.4.2 Confounding variables

Data will be collected regarding specific confounding variables including:

- Gestation
- Mode of delivery
- Prolonged rupture of membranes
- Maternal health (e.g. maternal viral load and CD4 count)
- Breast health (e.g. mastitis and breastmilk viral load)

These will be used in the analysis to estimate the main determinants of post-natal transmission.

#### 10.4.3 Multiple births

MTCT studies usually only include the HIV status of the first born of multiple births. This study, however, is mainly concerned with post-natal transmission. Although maternal recall may be more difficult with multiple children, individual data will be collected and each child will be included as a separate unit with respect to analysis of post-natal transmission. However for analysis of in utero and intrapartum transmission data, mother:multiple infant pairs, born by normal vaginal delivery will be considered as a single unit (HIV status of first born child).

#### 10.4 4 Feeding practice definitions and rationale

Feeding practices of women and infants will be categorised for analysis. In recognition of feeding patterns other than EBF being adopted, the following rationale and definition will be applied.

Studies have shown that different types of breastfeeding practices are unequal in the benefits they produce. EBF has been shown to be superior to MBF in terms of growth. As a result, definitions of breastfeeding practices are based mostly on growth rates and not on the potential to cause damage to the bowel. No single definition therefore suits the purpose of the study. The study, however, requires categories of breastfeeding which distinguish between those which contain virus in breastmilk and other fluids or foods which may or may not damage the bowel. Additional consideration is needed to ensure that the categories should be easy to assess in the field, but also that more complex categories can be modelled later to establish a dose-related effect.

| **Exclusive breastfeeding**  **[EBF]** | Breastmilk +/- medicines only e.g. vitamins, drugs, mineral supplements, oral rehydration solution | *Reference*  *23* |
| --- | --- | --- |
| **Exclusive formula (Never breastfed)**  **[EFF]** | Non-human milks +/- other liquids with or without solids | *24* |
| **Mixed breastfeeding [MBF]** | Breastmilk + non-human milks and/or other liquids with or without solids | *25* |

#### 10.4.5 Temporary lapses

It is recognised that some mothers will temporarily lapse from EBF or EFF and yet comply with an exclusive pattern for the majority of the study. If a mother interrupts EBF or EFF for a short period she may remain in the feeding arm to which she self-selected. Lapses of water or formula but not food, as defined below are allowed. These mothers and infants will continue to be regarded as legitimate EBF subjects eligible for analysis. However the following designations will apply to possible sub-analyses.

| **EBF(a)** | No lapses. Breastfed only throughout the entire study period |
| --- | --- |
| **EBF(b)** | 1 lapse only in study period but thereafter resuming EBF |
| **EBF(c)** | 2-3 lapses in study period, but EBF for the remaining duration of the study |

A lapse represents one day when either water or formula milk is given. Porridge or other solid foods, even on one occasion, will represent non-compliance with the intervention and the infant will be regarded as MBF. Mothers who temporarily depart from EBF to give oral rehydration solution only, for a maximum period of 48 hours, will still be regarded as EBF if they resume EBF.

#### 10.4.6 Deaths and loss to follow-up

Information on the cause of death of children who die during the course of the study will be collected on Adverse event forms. For children living within the Demographic Surveillance Area (DSA), this will be compared with information collected by the Verbal autopsy team of the DSS and the likely cause of death determined i.e. HIV-related or HIV-unrelated. For children living outside the DSA, information will be sought from relatives and the same diagnostic algorithms used to allocate cause of death. Children who are lost to follow-up will be tracked within the District or by telephone outside the District to determine survival status.

Feeding and transmission data will be included up to the last home and clinic visit when feeding practices and blood samples were collected. Transmission data from samples collected at the last clinic visit will be related to feeding practices reported at the home visit 4 week earlier. Mortality data will be analysed using Kaplan-Meier survival tables.

## 10.5 Sample size calculation

The sample size is calculated to determine the HIV infection rate within a population of EBF infants as determined by HIV RNA PCR analysis of samples collected at different time points i.e. 6 and 22 weeks. If adherence rates after one year indicate that we are unlikely to achieve a cohort of sufficient size to allow the preferred analysis, further enrolment will be determined by an accepted operational period for the study. In this case, the primary analysis will focus on all mothers exposed to the intervention. The available sample frame in this situation will always be significantly greater than those who would have EBF.

#### 10.5.1 Approach to sample size calculation for HIV-infected women

1. This is an implementation cohort study in which the primary objective is to describe the HIV infection rate of infants from samples collected at 22 weeks and who are EBF until 18 weeks of age.
2. This rate will be compared against a baseline HIV infection rate in EBF infants, defined as the combined *in utero* and intrapartum transmission, and determined by the HIV infection rate of EBF infants from samples collected at 6 weeks.
3. HIV infection rates of infants who are EFF is not a suitable comparison in this study, since we believe only a small proportion of women will choose to EFF from birth.
4. An HIV infection rate in EBF infants at 22 weeks of age more than 5% greater than the HIV infection rate at 6 weeks of age will be taken as an arbitrary but significant increment for public health considerations.
5. If the hypothesis is correct, that EBF is protective, the sample size needs to be able to detect

a). HIV infection rate at 6 weeks of ~11% ± 2%, and b). HIV infection rate at 22 weeks of ~16% ± 2% within the population of infants who are EBF.

*Sample size calculation*

Using the above approach,

- To detect if the HIV infection rate{x} (of samples collected) at 6 weeks of infants who are EBF until 6 weeks = 11% ± 2% with 80% power and 95% confidence intervals then x = p(1-p) = 979

e^2^

where x = the number of infants required to EBF until 6 weeks

p = HIV infection rate to be detected, and,

e = standard error

- To detect if the HIV infection rate{y} (of samples collected) at 22 weeks of infants who are EBF until 18 weeks = 16% ± 2% with 80% power and 95% confidence intervals then y = p(1-p) = 1344

e^2^

where y = the number of infants required to EBF until 18 weeks

p = HIV infection rate to be detected, and,

e = standard error

- The difference between the HIV infection rate of EBF infants at 22 weeks and at 6 weeks {x} will be deduced and the confidence intervals reported.

It is estimated that ~ 65% mothers:infants enrolling in the study will complete 18 weeks EBF. Therefore to allow for loss to follow up and deviation from study protocol, if y = 0.65 N, then N = 1344 / 0.65 = **2068**.

#### 10.5.2 Approach to sample size calculation for EBF adherence rates HIV-infected vs. HIV-uninfected women

- - 1. The sample size is calculated to detect a significant difference in EBF adherence rates between HIV-infected and uninfected mothers.
    2. EBF adherence in HIV-infected mothers is assumed to be 65%, while in HIV-uninfected it is assumed to be 55% (reduced motivation)
    3. Based on these assumptions, 395 HIV-infected and uninfected mother:infant pairs are required to detect this difference with 80% power at the 95% significance level.

**We will therefore aim to recruit 2,100 HIV-infected women and 400 HIV-uninfected women.**

See also Appendix C - Notes prepared by Prof ML Newell in response to comments from the Wellcome Trust Population panel, January 2001.

## 10.6 Interim analyses and study reviews

#### 10.6.1 Interim analysis

One interim analysis will be conducted after half the required subjects are enrolled and their 22 week blood sample has been obtained and analysed for HIV DNA by PCR. The main outcomes that will be assessed are HIV infection rates within 72 hours of birth, and at 6 and 22 weeks. Data from this analysis will be given to the Study Steering Committee and Data Monitoring and Safety Committee (DMSC).

It is expected that NVP will reduce the combined in utero and intrapartum transmission to ~11%. In an EFF population, no further transmission would be expected. If the interim analysis reveals that the 22 week HIV infection rate is >25% in the mothers:infants designated EBF at 18 weeks, the DMSC will be asked to review the study progress and determine if continuing the study is justified or if further data is required.

If the 22 week transmission rate of all mothers:infants exposed to the BCSS intervention is >25%, then the DMSC will also be asked to review the study progress and determine if continuing the study is justified or if further data is required. This is based on the following rationale:

1. The BCSS intervention has failed to achieve adherence with the substantial of the study or,
2. EBF adherence has been achieved by the intervention but has not produced any reduction in transmission in the larger study population, or,
3. EBF adherence has produced such a marginal reduction in transmission that promotion of EBF to the individual or community is not deemed an effective intervention to reduce MTCT HIV.

#### 10.6.2 Operational reviews

Operational reviews of study routines including VCT uptake and enrolment rates will be conducted on a monthly basis after the first enrolment.

#### 10.6.3 Formal study reviews

Formal study reviews will be conducted at 6, 12, and 24 months after the first enrolment. VCT, enrolment rates and adherence to EBF will be estimated at each of these times and combined to determine the impact on the required enrolment period and study duration. The data from the 12 and 24 month reviews will be referred to the Study Steering Committee and the DMSC.

# 11. Study management

## 11.1 Study Steering Committee

A Study Steering Committee (SSC) has been established to monitor and supervise the progress of the study towards the primary and secondary objectives. It will review at regular intervals relevant information from the study and also from other studies. It will also consider the recommendations of the Data Monitoring and Safety Committee.

## 11.2 Data Monitoring and Safety Board (DMSC)

A Data Monitoring and Safety committee (DMSC) has been established to consider data from the interim analyses, plus any additional safety issues and other relevant information from other sources. It will also determine if additional interim analyses of the study data should be undertaken and consider requests for the release of interim data and to recommend the advisability of this to the SSC. It will meet 2-3 times annually and monitor recruitment information, adverse event reports and other data from the interim analysis.

## 11.3 Study discontinuation

Any decision to stop the study will be taken only after discussion between the SSC and DMSC. The following are suggested as possible grounds for stopping.

1. If the 22 week HIV infection rate of infants designated EBF or the 22 week HIV infection rate of all mothers:infants exposed to the BCSS intervention is >5% (with 99% confidence intervals) more than the 6 week HIV infection rate at any interim analysis.
2. If the 22 week EBF adherence rate is <30% at the 12 month review or <40% at the 24 month review.
3. Enrolment rates are less than 50% at the 12 month review.

## 11.4 Confidentiality

All records will be stored at the Africa Centre to protect confidentiality. Personal data will be entered using a unique study number which will also be used and recorded on clinic cards and questionnaires. This ‘external’ identifier and personal characteristics allowing identification e.g. name, date of birth and residency, will be stored separately and can only be linked by authorised staff. An internal study number will be allocated by computer methods and linked to the external study number. All clinical data will be identified and analysed using this internal number. This system will protect personal information and clinical data. HIV results will be identified via a bar code system as described above, and will be linked (by computer) to the clinical data.

## 11.5 Biohazard risks

Standard University of Natal (UND) procedures for the disposal of all needles, blood, and blood products will be implemented for all field and clinic blood sampling. All used products will be placed in marked Biohazard boxes and returned to Durban for autoclaving and disposal. Staff will be given formal training in sample collection and safe disposal of products. In the event of a needlestick injury, standard UND policy will be adopted. Staff will be required to submit themselves to an immediate HIV test and if negative will be offered standard UND antiretroviral prophylaxis.

## 11.6 Ethical approval

The Ethics Committee of the University of Natal approved the study in August 2001. In addition, the Africa Centre has established a local Community Advisory Board (CAB) to provide an effective communication and consultative mechanism with representatives of the Mpukunyoni and Hlabisa tribal areas – the areas in which one arm of the study will be performed. Similar communication networks have been established with the Cato Manor Development Association (CMDA). Details and implications of the study have been discussed in full with the CAB/CMDA and regular meetings will provide opportunity for each to relate concerns and problems arising from the respective communities.

## 11.7 Timelines

- Recruitment period: September 2001 – March 2004
- Finishing date: October 2005
- Reporting date: March 2006 (or sooner, depending on what results are found)

# Appendix A. Consent and Patient information forms

Mamanengane - Africa Centre

##### CTT - Consent to test for HIV

I understand the benefits and difficulties of testing for HIV and also the implications for my family and myself. I understand that the test is voluntary. I understand that if I choose not to test then I am still free to attend the clinic and receive all the services as usual.

I have received a date to return to see the counsellor to hear my results.

Signature: _________________________________

Name: ____________________________________

Date: _______/ __________/ ______________

##### Witness

Signature: _________________________________

Name: ____________________________________

Date: _______/ __________/ ______________

Mamanengane - Africa Centre

##### CTE - Consent to enrol

I, ______________________________ agree to participate in the research study ***Mamanengane***. I have received and understood the study information sheet. I have discussed the advantages and disadvantages of participating in the study. I understand that the Mamanengane study team will visit me regularly at home. I agree to attend the clinics as explained in the information sheet. I agree to give samples of breastmilk and blood and for blood samples to be taken from my baby. I agree for the samples to be analysed for some tests immediately and to be stored for other tests later.

I know that I can leave the research study at any time without prejudice. I will still be able to attend and make full use of all the facilities at the health clinic as usual.

Signature: _________________________________

Name: ____________________________________

Date: _______/ __________/ ______________

##### Witness

Signature: _________________________________

Name: ____________________________________

Date: _______/ __________/ ______________

##### Witness

Signature: _________________________________

Name: ____________________________________

Date: _______/ __________/ ______________

You may keep one copy of this form. The other copy will remain in our study files.

**Mamanengane - Africa Centre**

**MAMANENGANE - Study Information Sheet**

**‘Mamanegane’ – a research study.**

We are asking you and your child, who will soon be born, to participate in a research study called Mamanengane, which will be conducted by the Africa Centre. A research study is the way for us to learn the answer to a problem.

**What are we trying to learn?**

In Mamanengane we want to learn what is the best way to feed young children over the first two years of life to improve their chances of survival. If a mother herself has HIV infection we want to see if exclusive breastfeeding prevents her child getting HIV. If a mother does not have HIV infection we want to see if giving breastmilk exclusively protects her child against infections such as diarrhoea or pneumonia and helps her child to grow better than children who are fed in other ways. In Mamanengane, there will be mothers who have HIV and also those who do not have HIV.

**Why is this important?**

This is important because many mothers in South Africa now have HIV infection. Also, many mothers give their children mixed feeds who then get diarrhoea and other serious infections. In the study, there will be mothers who both have HIV and also those who do not have HIV. Only the HIV counsellor and the study nurse will know these details.

**What we suggest about the way you feed your child.**

In Mamanengane, if a woman plans to breastfeed, then we suggest that she exclusively breastfeeds her child. By this we mean that she gives no other milk or food along with breastmilk. If a women plans to give formula feeds or another replacement milk then we suggest that she does so exclusively and does not mix it with any breastmilk. We will help and support mothers in Mamanengane to do whatever they choose.

**Who can be involved in Mamanengane and how long will it last?**

We are inviting both mothers who have HIV infection and also mothers who not have HIV infection to participate in Mamanengane. Only the HIV counsellor and the Mamanengane nurse will know these details. We hope that 2500 mothers and children will join Mamanengane. Mamanengane will continue in Hlabisa District for about 4 years.

**What will it mean if you participate in Mamanengane?**

1. If you agree to participate in the Mamanengane, we will visit you at your home before the birth of your baby and talk more about the study and what will happen each week. You and your family will be able to ask more questions at that time. We will also talk about your health e.g. your nutrition, family planning and avoiding sexually transmitted diseases.
2. **If you plan to breastfeed** –
3. When your baby is born one of our breastfeeding counsellors will visit you at the hospital or clinic and help you to start breastfeeding.
4. After your baby is born one of the breastfeeding counsellors will visit you at home every week for 2-3 weeks until you are comfortable with breastfeeding.
5. She will continue to visit you regularly every 2-3 weeks until your child is 6 months old.
6. **If you plan to give a replacement milk e.g. formula milk** –
7. We will discuss with you before delivery what is the safest way to prepare and store the replacement milk you choose.
8. We will discuss this again with you at the clinic after delivery.
9. Whether you breastfeed or give another milk, another field worker will also visit you at home every week until your baby is 6 months old. She will ask how you are feeding your baby and whether (s)he is having any health problems such as diarrhoea or pneumonia.
10. She will bring you a simple diary to record how you are feeding your child.

**What will we ask you to do?**

1. We will ask you to keep a simple diary to record how you are feeding your child and to record if you or your child have been unwell.
2. We will ask you to visit the clinic every month for the first 9 months. At the clinic we will weigh and measure you and your child and arrange immunisations for your child.
3. After your baby is 9 months old we will ask you to come to the clinic every 3 months until (s)he is 2 years old.
4. We will take blood samples from your child ten times during the first 9 months. We will do this 9 times by pricking the finger or heel of your child. Each time we do this we will only take a few drops of blood. Only once we will do this using a needle and syringe. At this time we will take 1½ teaspoons of blood. After 9 months until 2 years of age we will take blood another five times. At these times we will do it by pricking the finger or heel.
5. We will ask you to express breastmilk, about 2 teaspoons, each time you visit the clinic and we will collect this into a small bottle. We will take blood samples from you at 6 weeks and 6 months after your child is born. Each time this will be about two teaspoons.

**What will happen to these samples?**

Some of the samples will be tested immediately and some will be stored for testing later. We will be able to give the results of the tests that are done immediately.

**How may you and your child benefit from being in Mamanengane?**

- - If you are breastfeeding we will visit you at home during the first 6 months and help you to breastfeed in the best way and avoid some of the difficulties which can arise.
  - If you are formula feeding, we will advise you at the clinic about the safest way to do this.
  - If your child participates she or he may have less days of diarrhoea than other children because of better feeding.
  - Exclusive breastfeeding may also improve the growth and development of your child.
  - You will be able to see the Mamanengane nurse at the clinic if you have any difficulty.
  - We will be able to test your child and learn whether (s)he has HIV infection.
  - If your child has HIV infection we will give her/him medicine called cotrimoxazole to prevent other common infections such as pneumonia.

**Is there any disadvantage from being in Mamanengane?**

There will be no charge for participating in the study. Taking the blood samples may cause some pain at the time but this will stop after 1 or 2 minutes. There may be a bruise where the blood sample was taken.

**Will there be any charge for Mamanengane?**

There will be no charge for participating in Mamanengane.

**What if I cannot decide now or I change my mind later?**

The decision to participate or not in the Mamanengane is entirely yours. You do not have to decide now – you can talk this over with other family members. If you do not want to participate, you are free to say ‘no’. If you are unhappy later you can also leave the study without a problem. If you do not want to participate you will be free to attend the clinic as before and receive the same care.

**Who will see the information that we collect?**

All records will be kept completely confidential. Only senior members of the study team will see the results. Reports of Mamanengane will not give any names or personal details. We will inform the Hlabisa hospital and clinic staff and the local community about general results (not the results of any individual). We will also give the results to the KwaZulu Natal provincial authorities and also the national Department of Health.

We hope to have 2500 mother and children join the study. The study will continue for about 4 years in Hlabisa District. Once the study is completed we will inform you of the results of the study. We will also work closely with the District and Provincial Health Authorities to inform them of the results of the study.

**Who to contact if you want to know more, or if you have a problem at any time.**

If you want more information on **‘Mamanengane’** before deciding on whether or not to participate, or if you participate in **’Mamanengane’** and later have questions, please call:

**Dr Nigel Rollins, Africa Centre, Mtubatuba,**

**Tel: 035 550 0158**

**or,**

**Dr Ruth Bland, Africa Centre, Mtubatuba,**

**Tel: 035 550 0158**

If at any time you have questions about the rights of a person in a research study, please contact our Community Liason Office. The persons in the Community Liason Office are:

**Dudu Biyela, Community Liason Office, Africa Centre, Mtubatuba**

**Tel: 035 550 7532**

**or**

**Victoria Xolo, Community Liason Office, Africa Centre, Mtubatuba**

**Tel: 035 550 7531**

# Appendix B. UNAIDS recommendations on HIV and Infant feeding

# New Data on the Prevention of Mother-to-Child Transmission of HIV and their Policy Implications

# recommendations

# WHO Technical Consultation on Behalf of the UNFPA/UNICEF/WHO/UNAIDS Inter-Agency Task Team on Mother-to-Child Transmission of HIV

# Geneva, 11-13 October 2000

## Risks of breastfeeding and replacement feeding

- When replacement feeding is acceptable, feasible, affordable, sustainable and safe, avoidance of all breastfeeding by HIV-infected mothers is recommended.
- Otherwise, exclusive breastfeeding is recommended during the first months of life.
- To minimize HIV transmission risk, breastfeeding should be discontinued as soon as feasible, taking into account local circumstances, the individual woman’s situation and the risks of replacement feeding (including infections other than HIV and malnutrition).
- When HIV-infected mothers choose not to breastfeed from birth or stop breastfeeding later, they should be provided with specific guidance and support for at least the first 2 years of the child’s life to ensure adequate replacement feeding. Programmes should strive to improve conditions that will make replacement feeding safer for HIV-infected mothers and families.

## Cessation of breastfeeding

- HIV-infected mothers who breastfeed should be provided with specific guidance and support when they cease breastfeeding to avoid harmful nutritional and psychological consequences and to maintain breast health.

## Infant feeding counselling

- All HIV-infected mothers should receive counselling, which includes provision of general information about the risks and benefits of various infant feeding options, and specific guidance in selecting the option most likely to be suitable for their situation. Whatever a mother decides, she should be supported in her choice.
- Assessments should be conducted locally to identify the range of feeding options that are acceptable, feasible, affordable, sustainable and safe in a particular context.
- Information and education on mother-to-child transmission of HIV should be urgently directed to the general public, affected communities and families.
- Adequate numbers of people who can counsel HIV-infected women on infant feeding should be trained, deployed, supervised and supported. Such support should include updated training as new information and recommendations emerge.

## Breast health

- HIV-infected women who breastfeed should be assisted to ensure that they use a good breastfeeding technique to prevent these conditions, which should be treated promptly if they occur.

## Maternal health

- HIV-infected women should have access to information, follow-up clinical care and support, including family planning services and nutritional support. Family planning services are particularly important for HIV-infected women who are not breastfeeding.

# Appendix C. Statistical considerations regarding vertical transmission estimates

Prepared by Prof ML Newell in response to the Wellcome Trust Population panel, January 2001

Note VTR = Vertical transmission rate

1. The current sample size is estimated on the basis of precision estimate, assuming a rate of vertical transmission of 16% in the EBF group. To show a rate of vertical transmission at 22 weeks of 16%, with a precision of 1% on either side, the required sample size would be about 1350 women EBF up at least 18 weeks. Assuming 65% of women would do so this would mean 2076 women delivering live births. To show a vertical transmission rate at 22 weeks of 14% with 1% precision would require 1204 EBF women up to at least 18 weeks (and would need 1852 women at delivery); and to be confident in an 18% rate the number would have to be 1476 women (starting with 2270 women).

1. Assuming that the rate of VTR in the EBF group is 16%, which one would want to compare to an assumed VTR in MBF of 19%, with 80% power and 95% confidence interval of 5%, 1289 women would be needed (and 1983 women enrolling at delivery, of whom 65% EBF until 18 weeks).
2. To change the scenario to a simultaneous comparison between EBF and MBF, the following situations could arise:

65% EBF at 18 weeks, with a 16% VTR (at 22 weeks) and 35% MBF at 18 weeks with a 21% VTR (at 22 weeks) – estimated sample size would be 1457 EBF and 728 MBF at 22 weeks. If the VTR were 15% and 19% respectively but with a similar 2:1 ratio in the feeding groups, one would need 2122 MBF and 1061 EBF. In this case we would need to extend the enrolment period by 6 months. If the rates of vertical transmission were 12% in the EBF and 16% in the MBF and we had twice as many EBF than MBF, the numbers would have to be 1872 EBF versus 936 MBF.

If the ratio between EBF and MBF was 1:1, which would make us depressed with all the effort; and the rates of VTR were 16% and 21% respectively, there would have to be 985 women in each group. For VTRs of 15% and 19% in the EBF and MBF group, the numbers would have to be 1433 in each group.

I think this shows that it will be entirely feasible we will be able to have reliable results within a range of scenarios, and that if the worst comes to the worst we may need to extend the enrolment period by 6 months.

# References

1. Connor EM, Sperling RS, Gelber R et al. Reduction of maternal-infant transmission of Human Immunodeficiency Virus type 1 with zidovudine treatment. N Engl J Med 1994; 331: 1173.
2. Shaffer N, Chuachoowong R, Mock PA, et al. Short-course zidovudine for perinatal HIV-1 transmission in Bangkok, Thailand: a randomised controlled trial. Lancet 1999; 353: 773-80.
3. Wiktor SZ, Ekpini E, Karon JM, et al. Short-course oral zidovudine for prevention of mother-to-child transmission of HIV-1 in Abidjan, Côte d’Ivoire: a randomised trial. Lancet 1999; 353: 781-85.
4. Toussaint S, Ekpini ER, Nkengasong J et al. Efficacy of a short course oral zidovudine regimen administered in late pregnancy in preventing mother to child transmission of HIV_1_ in Abidjan, Côte d’Ivoire: Long term follow-up in a breastfeeding population. Poster 234. Second Conference on Global Strategies for the Prevention of HIV Transmission from Mothers to Infants. Sept. 1-6, 1999. Montreal, Canada.
5. Dabis F, Masellati P, Meda N, et al. 6-month efficacy, tolerance, and acceptability of a short regimen of oral zidovudine to reduce vertical transmission of HIV in breastfed children in Cote d’Ivoire and Burkina Faso : a double-blind placebo-controlled multicentre trial. Lancet 1999; 353: 786-92.
6. Ditrame ANRS 049 study group. 15 month efficacy of maternal oral zidovudine to decrease vertical transmission of HIV-1 in breastfed African children. Lancet 1999; 354:2050-2051.
7. Saba J. The results of the PETRA intervention trial to prevent perinatal transmission in sub-Saharan Africa. 6^th^ Conference on Retroviruses and Opportunistic Infections. Abstract S7. January 31-Februrary 4, 1999. Chicago, Ill, USA.
8. Guay LA, Musoke P, Fleming T, et al. Intrapartum and neonatal single-dose nevirapine compared with zidovudine for prevention of mother-to-child transmission of HIV-1 in Kampala, Uganda: HIVNET 012 randomised trial. Lancet 1999; 354: 795-802.
9. Owor M, Deseyve M, Duefield C, Jackson B. The one year safety and efficacy data of the HIVNET 012 trial. Abstract LbOr1. XIII^th^ International Aids Conference. 9-14 July, 2000. Durban, S. Africa.
10. Gray G. The PETRA study: early and late efficacy of three short ZDV/3TC combination regimens to prevent mother to child transmission of HIV-1. Abstract LbOr5. XIII^th^ International Aids Conference. 9-14 July, 2000. Durban, S. Africa.
11. Moodley, D. The SAINT trial: Nevirapine vs. zidovudine (ZDV) and lamivudine (3TC) in prevention of peripartum HIV transmission. Abstract LbOr2. XIII^th^ International Aids Conference. 9-14 July, 2000. Durban, S. Africa
12. Dunn DT, Brandt CD, Krivine A et al. The sensitivity of HIV_1_ DNA polymerase chain reaction in the neonatal period and the relative contribution of intra-uterine and intra-partum transmission. AIDS 1995; 9:F7-11.
13. UNAIDS. HIV and Infant Feeding. A review of HIV transmission through breastfeeding. UNAIDS/WHO Joint United Nations Programme on HIV/AIDS (UNAIDS). World Health Organisation. Geneva. June 1998.
14. Coutsoudis A, Pillay K, Spooner E, et al. Influence of infant feeding patterns on early Mother-to-Child transmission of HIV_1_ in Durban, South Africa: a prospective cohort study. Abstract LbOr6. XIII^th^ International Aids Conference. 9-14 July, 2000. Durban, S. Africa
15. Coutsoudis A, Pillay K, Spooner E et al. Randomised trial testing the effect of Vitamin A supplementation on pregnancy outcomes and early Mother-to-Child HIV_1_ transmission in Durban, South Africa. AIDS 1999; 13: 1517-1524.
16. Morrow AL, Guerrero ML, Shults J, et al. Efficacy of home-based peer counselling to promote exclusive breastfeeding: a randomised controlled trial. Lancet 1999; 353: 1226-1231.
17. Bobat R, Moodley D, Coutsoudis A, Coovadia HM. Breastfeeding by HIV_1_-infected women and outcome in their infants: a cohort study from Durban, South Africa. AIDS 1997; 11:1627-1633.
18. Grummer-Strawn LM, Rice SP, Dugas K, Clark LD, Benton-Davis S. An evaluation of breastfeeding promotion through peer counselling in Mississippi WIC clinics. Maternal Child Health J 1997; 1:35-42
19. Haider R, Islam A, Hamadani JD, Amin NJ, Kabir I, Malek MA, Mahalanabis D, Habte D. Breast-feeding counselling in a diarrhoeal disease hospital. Bull WHO 1996; 74(2): 173-179.
20. Haider R, Ashworth A, Kabir I, Huttly SRA. Peer counsellors improve early post partum breastfeeding practices in an urban community in Dhaka, Bangladesh. Proceedings of the Nutrition Society 1998; 57:63A.
21. Green CP. Improving breastfeeding behaviours: Evidence from 2 decades of intervention research. Linkages project. Academy for Educational Development, Washington DC, USA 1999.
22. Nicoll A, Newell M-L, van Praag E et al. Infant feeding policy and practice in the presence of HIV_1_ infection. AIDS 1995; 9: 107-19.
23. Nduati R. Clinical Studies of Breast vs. Formula Feeding. Second Conference on Global Strategies for the Prevention of HIV Transmission from Mothers to Infants. Sept. 1-6, 1999. Montreal, Canada.
24. WHO. Indicators of assessing breast-feeding practices. Report of an Informal Meeting. 11-12 June, 1991. Geneva. WHO.
25. Labbock M, Krasovec K. Toward consistency in breastfeeding definition. Stud Fam Plann 1990; 21: 226-30.
26. Piwoz EG, de Kanashiro HC, de Romana GL et al. Feeding practices and growth among low-income Peruvian infants: a comparison of internationally-recommended definitions. Int J Epidem 1996; 25: 103-114.
27. Miotti PG, Taha TFT, Kumwenda NI et al. HIV Transmission through breastfeeding. JAMA 1999; 282: 744-749.
